# Implementing a multidimensional faculty promotion matrix at Saint George University of Beirut Faculty of Medicine

**DOI:** 10.12688/mep.20416.1

**Published:** 2024-08-27

**Authors:** Alexandre Nehme, Carmen El Khoury, Marc Jreij, George Karam, Ghewa El Achkar, Ziad Tannous

**Affiliations:** 1 Faculty of Medicine, Saint George University of Beirut, Beirut, Lebanon; 2 Saint George Hospital University Medical Center, Beirut, Beirut Governorate, Lebanon

**Keywords:** Faculty Promotion, Multidimensional Evaluation, Academic Metrics, Medical Education, Institutional Development, Performance Assessment, Faculty Development Programs

## Abstract

This paper outlines the development and implementation of a multidimensional faculty promotion matrix at Saint George University of Beirut Faculty of Medicine (SGUB FM). The matrix, designed to provide a comprehensive and equitable evaluation of faculty across multiple dimensions, is anchored in six pillars: Research, Clinical Practice, Teaching Effort, Administrative Effort, Community Work, and Extra Degrees and Awards. These pillars encompass diverse components, including publication output, clinical activities, teaching responsibilities, administrative roles, community engagement, and additional qualifications, with each metric normalized using z-scores for fair comparison.

This matrix analyzed the CVs and relevant documents of 112 faculty members, demonstrating its efficacy in providing equitable evaluation regardless of gender or rank. The results showed no significant differences in promotion rates among various faculty ranks, highlighting the matrix’s fairness and impartiality. The study also explores the relationship between faculty ranks and various performance metrics, revealing patterns in research productivity, clinical practice, and community engagement that escalate with higher academic ranks.

SGUB FM's approach aligns its curricular designs and instructional implementations with international benchmarks, particularly those set by the Association of American Medical Colleges (AAMC), ensuring global standard compliance while catering to the institution's unique context. The matrix serves not only as an evaluation tool but also as a catalyst for faculty excellence and professional development. This case study offers valuable insights for medical institutions developing inclusive promotion criteria and emphasizes the importance of holistic evaluation frameworks in fostering academic excellence and professional growth.

## Introduction

Faculties of medicine worldwide encounter significant challenges in establishing comprehensive and multidimensional matrices that guide promotion processes. As emphasized by Salajegheh *et al.* (2022), robust promotion criteria are fundamental for creating standards that acknowledge faculty members based on their achievements across a spectrum of activities, including research, teaching, and clinical skills ( Dhulkhed *et al.*, 2016; Gusic *et al.*, 2014). However, a universal standard for promotions remains elusive, with institutions often developing criteria tailored to their unique needs. Historically, medical school promotions have heavily favored research productivity, prompting a reassessment of traditional metrics such as peer-reviewed papers and citations ( Dhulkhed *et al.*, 2016; Gusic *et al.*, 2014). There is an increasing emphasis on a holistic approach that incorporates teaching and clinical performance ( Rifai *et al.*, 2019). Teaching assessment has evolved to include multidimensional criteria that focus on fairness and comprehensive methods for faculty improvement thus reflecting the faculty member's contribution to the overall quality of education. Teaching excellence acknowledges the value of scholarly activities, a significant component to be weighed in any promotion matrix ( Glassick, 2000; Ory, 2000).

This lack of Universal criteria presents a particular challenge for nascent medical schools, such as the Saint George University of Beirut Faculty of Medicine (SGUB FM), which lacks established research and previous teaching records as a benchmark for assessment. Founded amidst national turmoil and the global COVID-19 pandemic, SGUB FM welcomed its inaugural cohort of medical students in the fall of 2022. Despite these initial hurdles, the university has embraced innovative instructional strategies aligned with its curriculum, highlighting the need for a promotion system that reflects the diverse contributions of its clinical faculty ( Glassick, 2000; Ory, 2000).

This article explores the initial development and effectiveness of the promotion matrix used by SGUB FM during its early stages. By examining the matrix utilized by SGUB FM, this study offers valuable insights for other emerging faculties of medicine seeking to establish inclusive promotion criteria in the absence of established records. Such an approach not only facilitates benchmarking with regional and international institutions but also reaffirms a commitment to objective and valid promotion processes. This underscores the importance of balanced criteria in faculty promotions, accommodating both traditional metrics and the additional qualifications necessary for a comprehensive evaluation.

## Methods

### Ethical approval and consent

This study was conducted with the written approval of the Institutional Review Board (IRB) at Saint George University of Beirut Faculty of Medicine (SGUB FM). The ethical approval was granted under the reference number IRB-REC/O/021-24/1124 issued on 19 June 2024.

***Participant consent***

All participants in this study provided informed consent prior to their participation. The consent process was conducted as follows:

1   Type of Consent: Written consent was obtained from all participants.

2   Consent Process: Participants were provided with a detailed information sheet outlining the study's purpose, procedures, potential risks, and benefits. They were given the opportunity to ask questions and were assured of their right to withdraw from the study at any time without any consequences.

The ethical guidelines followed in this study adhered to the principles set forth in the Declaration of Helsinki and the regulations established by the SGUB FM IRB. The anonymity and confidentiality of the participants were maintained throughout the study, and all data were securely stored and accessed only by authorized personnel.

### Development of the scoring matrix

The Faculty Affairs Committee at Saint George University of Beirut Faculty of Medicine (SGUB FM) has meticulously devised a comprehensive scoring matrix for academic promotions, anchored in the principles set out by the American Association of Medical Colleges (AAMC) ( Gusic *et al.*, 2014). This matrix, a cornerstone of SGUB FM's Appointment, Reappointment, and Promotion Policy, thoroughly assesses faculty members across six essential dimensions or pillars: research, clinical practice, teaching efforts, administrative roles, community engagement, and additional qualifications, including extra degrees or awards. This multifaceted approach ensures a holistic and balanced evaluation of the faculty's diverse contributions.

The promotion process at SGUB FM is deeply intertwined with its historical roots as a complementary entity to the Saint George Hospital University Medical Center (SGHUMC), Lebanon's oldest teaching medical facility, founded in 1878. Initially, the University of Balamand (UOB), affiliated with SGHUMC, handled the assignment of faculty ranks. With the creation of SGUB FM, a strategic choice was made to maintain the faculty titles awarded by UOB, such as instructor, assistant professor, associate professor, and full professor. SGUB FM's approach to faculty promotion respects these pre-existing ranks, smoothly transitioning from the University of Balamand's system.

During its formative years, SGUB FM strategically acknowledged previously granted ranks and assessed their eligibility for promotion within its own matrix. This method reflects a deliberate integration of historical recognition with the dynamic academic demands of SGUB FM.

### Scoring criteria and calculation method

The scoring matrix is based on a comprehensive evaluation sheet that incorporates all metrics across the six pillars. To standardize and compare diverse metrics, a normalization employing the z-score calculation was computed for each data point ( Calandro, 2007). The z-score, or standard score, is a statistical measurement representing the number of standard deviations a data point is from the mean of a dataset. This normalization facilitates comparison across different distributions. All the clinical faculty members that applied for promotion were grouped as per their current exiting ranks yielding to three categories, the instructor category, the assistant professor category, and the associate professor category.

The formula employed for computing the z-score for each metric within each of the six evaluation pillars is *Z*=( *X*− *μ*)/ *σ*, where *Z* is the z-score, *X* is the data point, *μ* is the mean of the dataset, and *σ* is the standard deviation. The mean for our dataset was set at the international standard of the 50th percentile, with a correlated standard deviation of 10.

### Pillars of the scoring matrix ( Table 1)

**Table 1.** Scoring Matrix table summary.

#	Pillar	Description	Formula
**1**	**Research Pillar**	Evaluates research output and impact using publication metrics and academic influence. Includes Total Number of Publications, Recent Publications, Adjusted Impact Factor Per Author and Article, and H-index.	Research Pillar Score = (Total Publications Z-Score×0.20) + (RP Z-Score×0.10) + (AIFAP Z-Score×0.25) + (AIFA Z- Score×0.25) + (H-index Z-Score×0.20)
**2**	**Clinical Practice** ** Pillar (CP)**	Measures clinical performance at SGHUMC using metrics like Number of Inpatient Discharges, Inpatient Consultations, and Labs for Outpatients.	Clinical Practice Pillar Score = (ID2021 Z-Score×0.40) + (IC2021 Z-Score×0.20) + (OH2021 Z-Score×0.40)
**3**	**Teaching Effort ** **Pillar (TE)**	Focuses on instructional roles at SGUB FM, assessing overall teaching effectiveness.	Teaching Effort Pillar Score = TE Z-Score×1.00
**4**	**Administrative** ** Effort Pillar (AE)**	Evaluates engagement in clinical and academic administration at SGHUMC and SGUB, measuring hours spent in various administrative roles.	Administrative Effort Pillar Score = (CA Z-Score×0.35) + (AA Z-Score×0.35) + (CCA Z-Score×0.30)
**5**	**Community Work ** **Pillar (CW)**	Assesses engagement in community-oriented activities, using a rubric that evaluates the scope, leadership, impact, commitment, and collaboration in community work.	Community Work Pillar Score = (Scope of Involvement Z-Score x 0.20) + (Leadership & Initiative Z-Score x 0.20) + (Impact & Results Z-Score x 0.20) + (Commitment & Duration Z-Score x 0.20) + (Collaboration & Networking Z-Score x 0.20)
**6**	**Extra Degrees and** ** Awards Pillar**	Recognizes additional qualifications and awards, counting extra medical and non-medical degrees, and awards received.	Extra Degrees and Awards Pillar Score = (Extra Medical Degrees Z-Score×0.40) + (Non-Medical Degrees Z- Score×0.40) + (Awards Z-Score×0.20)

1. **Research Pillar**: The Research Pillar in our faculty evaluation framework quantifies and appreciates faculty members' research output and impact, primarily through publication metrics and their academic influence. It encompasses:

- Total Number of Publications - Counts the cumulative number of published works, constituting 20% of the Research Pillar’s weight.
- Recent Publications (RP) - Assesses publications from the last 7 years, contributing 10% to the pillar’s weight, highlighting recent research activity.
- Adjusted Impact Factor Per Author (AIFAP) and Per Article (AIFA) - These metrics, each comprising 25% of the pillar’s weight, evaluate the impact of an author's and individual articles' contributions. The adjustment considers citations, author count, publication year, and the journals' field-normalized impact.
- H-index (H) - Measures productivity and citation impact of scholarly works, accounting for 20% of the pillar's weight.

Data from Scopus and Web of Science are used for analysis. The Research Pillar contributes 30% to the overall faculty score. The overall pillar score is calculated using a weighted average of the z-scores for these metrics, following this formula:

Research Pillar Score = (Total Publications Z-Score×0.20) +(RP Z-Score×0.10)+(AIFAP Z-Score×0.25)+(AIFA Z-Score×0.25)+(H-index Z-Score×0.20)

2. **Clinical Practice Pillar (CP)**: The CP pillar in our faculty evaluation framework is centered around clinical activities of faculty members at SGHUMC. This pillar measures performance based on three main metrics as of 2021:

- ID2021: Number of Inpatient Discharges - This metric, constituting 40% of the CP pillar’s weight, tracks the number of patients discharged.
- IC2021: Number of Inpatient Consultations - Accounting for 20% of the pillar's weight, this metric assesses the number of consultations conducted with inpatients.
- OH2021: Number of Labs for Outpatients - This component represents 40% of the CP pillar's weight, focusing on the number of laboratory tests conducted for outpatients.

The data for these metrics is sourced from the Chief Medical Officer (CMO) office at SGHUMC. The Clinical Practice Pillar contributes 25% to the overall faculty score. The score for this pillar is calculated using a weighted average of the z-scores for each metric. The formula for this calculation, reflecting the stated percentages, is: Clinical Practice Pillar Score= (ID2021 Z-Score×0.40) +(IC2021 Z-Score×0.20)+(OH2021 Z-Score×0.40)

3. **Teaching Effort Pillar (TE):**The TE pillar in our faculty evaluation framework focuses on instructional roles at SGUB FM as of 2021, emphasizing curriculum development, classroom teaching, and the assessment of teaching effectiveness. This pillar constitutes 100% of the TE category and contributes 15% to the overall faculty score. The score for the Teaching Effort Pillar is calculated using the TE Z-Score, reflecting the comprehensive nature of teaching responsibilities: Teaching Effort Pillar Score=TE Z-Score×1.00

4. **Administrative Effort Pillar (AE)**: The AE pillar in our faculty evaluation framework evaluates faculty members' engagement in clinical and academic administration at SGHUMC and SGUB. This pillar accounts for the time dedicated to various administrative roles and committee memberships, reflecting the scope of each faculty member's administrative involvement within the institution. The AE Pillar comprises three distinct components:

- CA: The number of hours spent in clinical administration at SGHUMC, contributing 35% to the AE pillar's weight retrieved from the CMO office.
- AA: The number of hours dedicated to academic administration at SGUB, also accounting for 35% of this pillar's weight.
- CCA: The time spent in higher administrative capacities such as chief or chairman roles at both SGHUMC and SGUB, constituting 30% of the AE pillar's weight.

Collectively, these components represent a comprehensive view of a faculty member's administrative contributions. The Administrative Effort Pillar contributes 15% to the overall faculty score. The score for the AE Pillar is calculated using a weighted average of the z-scores for each metric. The formula, incorporating the specified percentages, is as follows:

Administrative Effort Pillar Score= (CA Z-Score×0.35)+(AA Z-Score×0.35)+(CCA Z-Score×0.30)

5. **Community Work Pillar (CW)**: The CW Pillar in our faculty evaluation framework assesses faculty members' engagement in broader community-oriented activities and was self-reported by each applicant in the CV. This pillar captures involvement in non-governmental organizations (NGOs), ministerial committees, and national or international scientific committees. Evaluation within this pillar is multifaceted, encompassing both the depth and breadth of participation in these organizations.

To effectively quantify these contributions, a specialized rubric (table…), accessible at Community Work Rubric, has been employed. This rubric segments the evaluation into five key areas, each equally weighted to contribute 20% of the total CW score:

- Scope of Involvement
- Leadership & Initiative
- Impact & Results
- Commitment & Duration
- Collaboration & Networking

These components collectively recognize the varied dimensions of community work. Faculty members self-report their community involvement activities, which are then documented in their CVs for assessment purposes. The Community Work Pillar constitutes 5% of the overall faculty score. The overall score for the CW Pillar is calculated as a weighted average of the z-scores for these metrics, following the distribution outlined in the rubric. The formula is:

Community Work Pillar Score = (Scope of Involvement Z-Score x 0.20) + (Leadership & Initiative Z-Score x 0.20) + (Impact & Results Z-Score x 0.20) + (Commitment & Duration Z-Score x 0.20) + (Collaboration & Networking Z-Score x 0.20)

6. **Extra Degrees and Awards Pillar**: The Extra Degrees and Awards Pillar in our faculty evaluation framework is designed to recognize and quantify the additional qualifications and recognitions achieved by faculty members. This data was self-reported by the applicant through the CV. This pillar encompasses:

- Extra Medical Degrees after Fellowship - Each additional medical specialty training acquired post-fellowship is assigned a value of 1 and contributes 40% to the weight of this pillar.
- Non-Medical Degrees - Similar to medical degrees, each non-medical degree attained is valued at 1, also accounting for 40% of this pillar's weight.
- Awards - Each award received is assigned a value of 1, contributing 20% to the pillar's weight.

The evaluation within this pillar thus sums up the total number of extra degrees (both medical and non-medical) and awards, each metric contributing a designated percentage to the pillar's overall score. The Extra Degrees and Awards Pillar constitutes 10% of the overall faculty score. To calculate the score for this pillar, a weighted average of the z-scores for the metrics of extra degrees and awards is used. The formula, based on the specified weightings, is:

Extra Degrees and Awards Pillar Score=(Extra Medical Degrees Z-Score×0.40)+(Non-Medical Degrees Z-Score×0.40)+(Awards Z-Score×0.20)

Including the extra awards and additional degrees pillar in our promotion criteria for faculty members is crucial for recognizing their achievements, promoting diversity and innovation, enhancing institutional reputation, attracting and retaining talent, and encouraging lifelong learning. It acknowledges exceptional accomplishments, fosters a multidisciplinary approach, enhances credibility, and motivates continued professional development.

### Overall score calculation ( Table 2)

**Table 2.** Overall Score Calculation table summary.

#	Pillar	Weight in Total Score	Description
**1**	**Research Pillar**	30%	The score is calculated from publication metrics and impact, contributing 30% to the overall score.
**2**	**Clinical Practice Pillar (CP)**	25%	This pillar assesses clinical activities and contributes 25% to the total score.
**3**	**Teaching Effort Pillar (TE)**	15%	Focuses on teaching roles and effectiveness, representing 15% of the overall score.
**4**	**Administrative Effort Pillar** ** (AE)**	15%	Evaluates administrative roles within the faculty, accounting for 15% of the total score.
**5**	**Community Work Pillar (CW)**	5%	Measures community engagement and activities, making up 5% of the overall score.
**6**	**Extra Degrees and Awards** ** Pillar**	10%	Recognizes additional qualifications and achievements, contributing 10% to the total score.

The overall score for each candidate is determined by a weighted summation of the scores from each pillar, where each pillar's score is itself a weighted sum of its constituent metrics’ z-scores. The weight distribution for each pillar in the total score is as follows: Research (30%), Clinical Practice (25%), Teaching Effort (15%), Administrative Effort (15%), Community Work (5%), and Extra Degrees and Awards (10%). Given this, the formula for the overall score is:

Overall Score= (Research Pillar Score×0.30)+(Clinical Practice Pillar Score×0.25)+(Teaching Effort Pillar Score×0.15)+(Administrative Effort Pillar Score×0.15)+(Community Work Pillar Score×0.05)+(Extra Degrees and Awards Pillar Score×0.10)

This calculation ensures that the final score comprehensively reflects the candidate's performance across all the specified domains. Both Excel 365 and SPSS version 27 were used for calculations and statistics to ensure accuracy and reliability in the scoring process.

### Prefix/suffix calculation

In alignment with the Appointment reappointment promotion policy (ARPP) policy, the score combining the research and education pillars is calculated against the total score to determine the prefix (for scores above 20%) and suffix (for scores below 20%).

- **Prefix**: In the scoring system, a prefix is added to the title of a faculty member when the combined score from the research and teaching pillars constitutes over 20% of their overall score. This prefix serves as a marker of the faculty member's strong focus and noteworthy achievements in research and education. It designates them as particularly adept in these areas, highlighting their dedication to advancing academic knowledge and teaching.
- **Suffix**: On the other hand, a suffix is attached to the title of a faculty member when their combined score from the research and teaching pillars is less than 20% of the overall score. This suffix indicates a greater dedication to clinical tasks and responsibilities. It signifies that the faculty member’s primary contributions and strengths lie in the clinical aspect of their role, emphasizing their commitment to patient care and clinical services. The suffix helps differentiate faculty members who are more clinically oriented from those who are more research and education focused.

## Results and analysis

**Figure 1.**
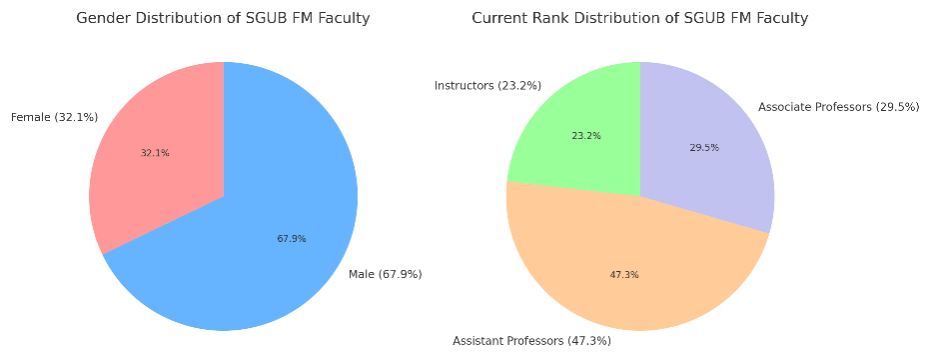
Gender and Rank Distribution and of SGUB FM Faculty.

The promotion matrix of Saint George University of Beirut Faculty of Medicine (SGUB FM) was evaluated across a dataset of 112 faculty members that responded to the call for CV submission sent by the Faculty Affairs committee to all faculty members at SGUB FM/SGHUMC, revealing a comprehensive assessment without any missing entries. The faculty distribution included 36 female members (32.1%) and 76 male members (67.9%) ( Figure 1), evidencing a male-dominant faculty body. The year of last promotion across this dataset averaged at 2010, indicating a mix of recently promoted individuals and those with more extended tenures at the institution. The current ranks within this dataset are comprised of 26 instructors (23.2%), 53 assistant professors (47.3%), and 33 associate professors (29.5%) ( Figure 1).

### Section I: normalized evaluation of faculty achievement using z-scores

**Figure 2.**
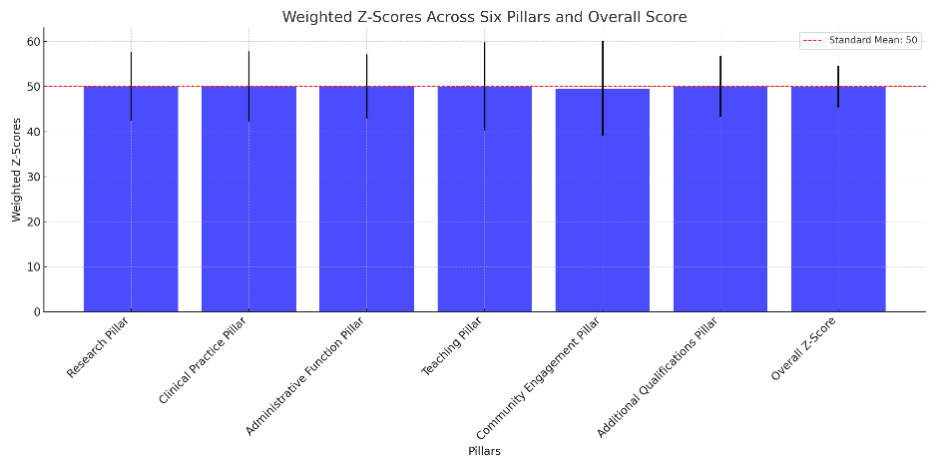
Weighted Z-score mean across the six pillars.

**Figure 3.**
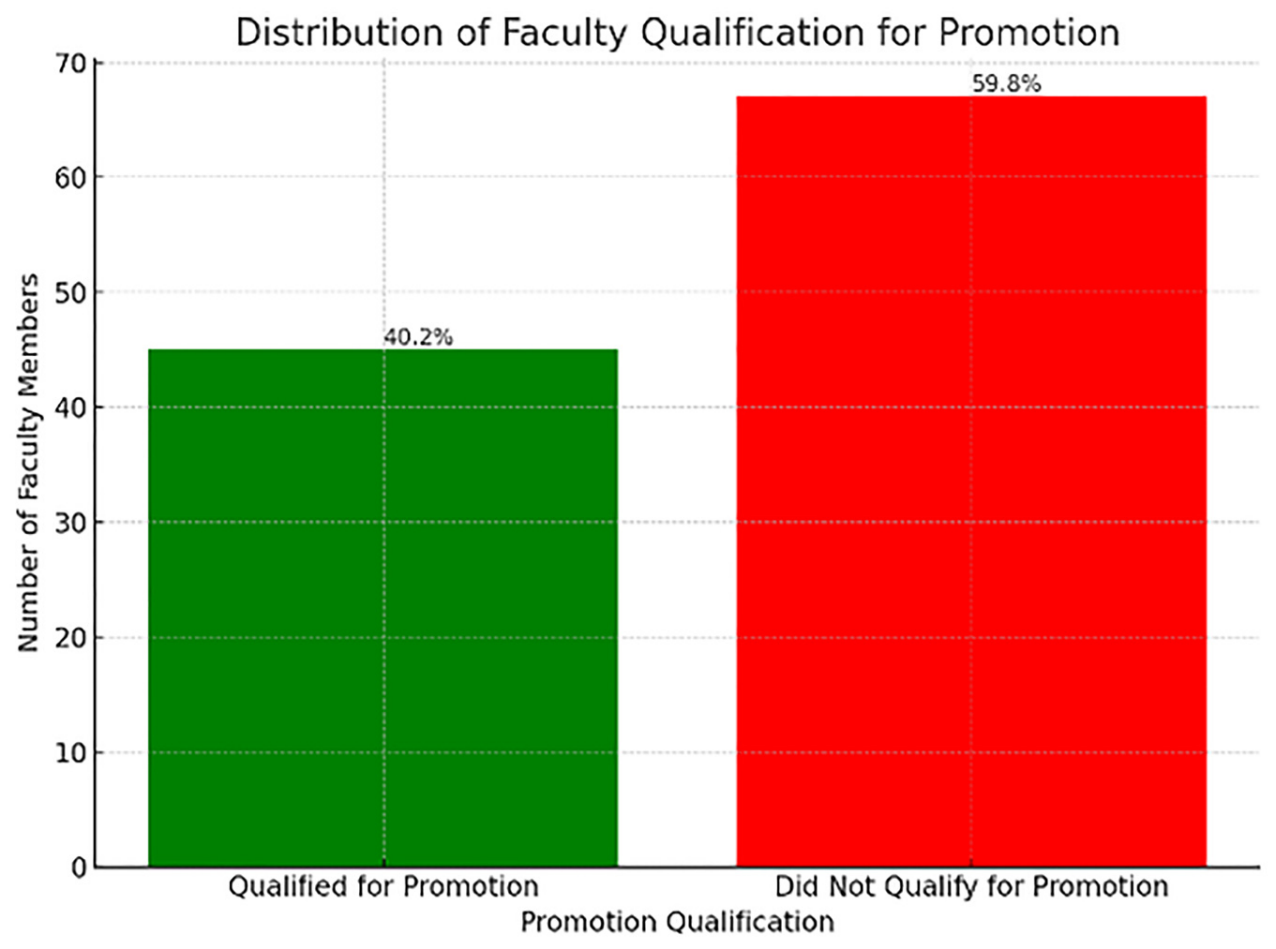
Percentage of Faculty Qualified for Promotion.

The descriptive statistics for the weighted z-scores of the six pillars reveal a consistent evaluation across 112 faculty members ( Figure 2 and Figure 3).

1. **Research Pillar**: The weighted z-scores for Research ranged from 39 to 81, with an average score of 50.03, closely aligning with the standardized mean of 50. This pillar showed a standard deviation of 7.699, indicating a moderate spread around the mean score ( Figure 2).
2. **Clinical Practice Pillar**: Faculty performance in Clinical Practice also centered around the standardized mean with scores extending from 42 to 81, and an exact average of 50.00. The standard deviation here was 7.792, suggesting a comparable variability in clinical contributions to the Research pillar ( Figure 2).
3. **Administrative Function Pillar**: For Administrative Functions, the scores varied from 39 to 82, maintaining a mean of 50.00. A slightly lower standard deviation of 7.162 compared to other pillars indicates a some what tighter cluster of scores around the mean ( Figure 2).
4. **Teaching Pillar**: The Teaching scores spanned a wider range, from 42 to 98, with the mean being slightly below the standardized mean at 49.96. This pillar had a higher standard deviation of 9.827, reflecting the largest variability in z-scores among the pillars ( Figure 2).
5. **Community Engagement Pillar**: Community involvement displayed the broadest range of scores from 1 to 78, with a mean of 49.53. The substantial standard deviation of 10.546 underscores a significant diversity in community engagement activities among the faculty ( Figure 2).
6. **Additional qualifications Pillar**: The Extra pillar, which accounts for additional qualifications, showed scores ranging between 33 and 68, averaging exactly at 50.00 with a standard deviation of 6.789, the lowest among the pillars, indicating a more concentrated distribution of scores ( Figure 2).

**Weighted Overall Z-Score**: The aggregate of the weighted z-scores, which constitutes the Overall Z-Score, ranged from 42 to 64 with a near-standard mean of 49.98 and a standard deviation of 4.634. This suggests that when considering all pillars collectively, faculty performance is closely aligned around the mean, reflecting a balanced evaluation process ( Figure 2).

All results are based on the full count of valid cases (N=112), ensuring that the analysis is comprehensive and representative of the SGUB FM faculty ( Table 3). To categorize individuals into "qualified for promotion" (scores above 50) and "not qualified" (scores at or below 50), a new binary variable was created in SPSS named 'Final'. This categorization involved re coding the 'Total Score' variable such that scores below 50 were flagged as '0', indicating non-eligibility for promotion, and scores at and above 50 were flagged as '1', indicating eligibility.

**Table 3.** Metrics Z score descriptive statistics.

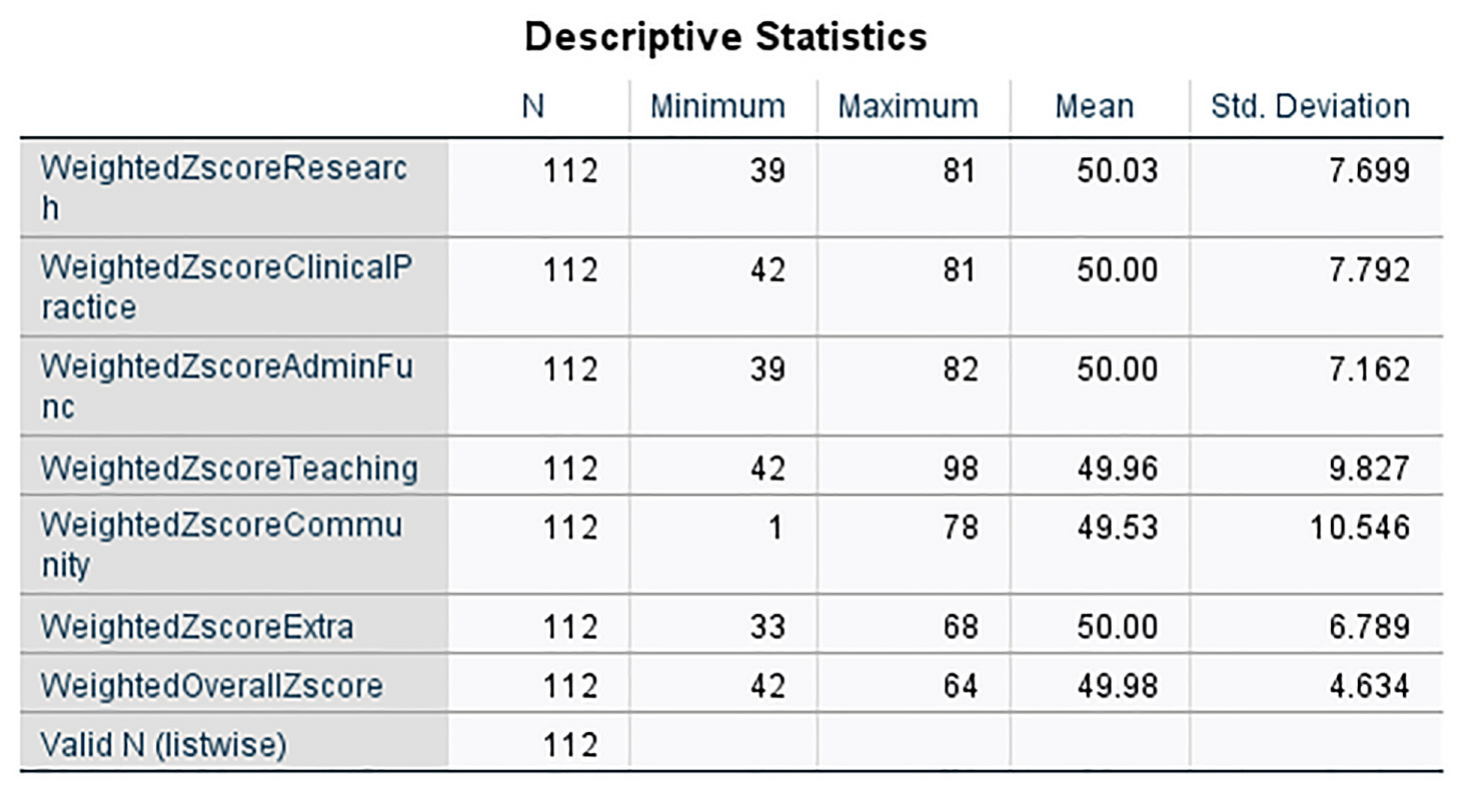

Following the recoding process, a frequency analysis of the 'Final' variable revealed that out of 112 faculty members, 45 individuals—accounting for 40.2% of the faculty—achieved scores of 50 and above, thus qualifying for promotion. Meanwhile, 67 faculty members, making up 59.8%, scored below the 50-point threshold and did not meet the criteria for promotion.

The crosstabulation and Chi-Square test results provide an overview of the promotion outcomes across different faculty ranks at Saint George University of Beirut Faculty of Medicine. The faculty ranks, coded as 1 (Instructors), 2 (Assistant Professors), and 3 (Associate Professors), were cross-tabulated against a binary promotion outcome where '1.00' indicates those who qualified for promotion and '.00' indicates those who did not.

**Figure 4.**
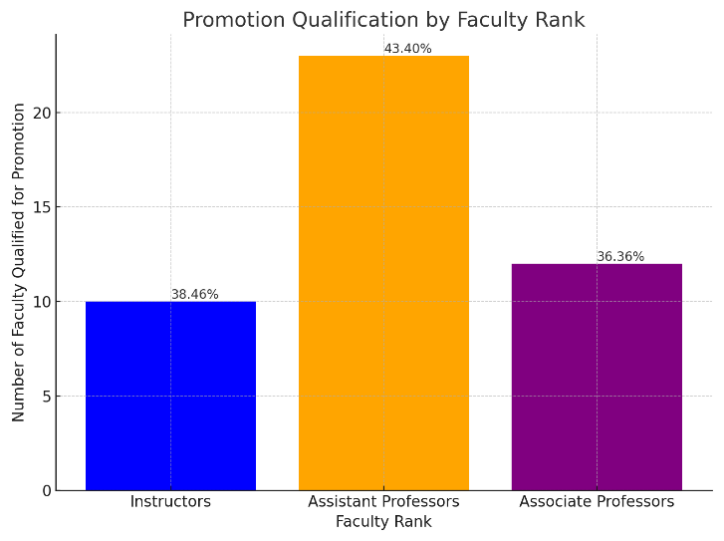
Percentage of Faculty Qualified for Promotion by rank.

The data indicates that the proportion of faculty meeting the criteria for promotion includes ( Figure 4):

- 10 out of 26 Instructors, representing roughly 38.46%,
- 23 out of 53 Assistant Professors, amounting to approximately 43.40%,
- 12 out of 33 Associate Professors, or about 36.36%.

However, the Chi-Square test of independence, with a Pearson Chi-Square value of .460 and a significance level (p-value) of .795, suggests that there is no statistically significant association between faculty rank and the likelihood of promotion. This is further supported by the Likelihood Ratio (p-value of .794) and the Linear-by-Linear Association (p-value of .829), both confirming the absence of a statistically significant trend across the ranks.

The number of valid cases was 112, and no expected cell counts were below 5, which satisfies one of the assumptions for the validity of the Chi-Square test.

In summary, the promotion process, when analyzed statistically, appears to be fair and unbiased across different faculty ranks, as there is no significant difference in promotion rates among Instructors, Assistant Professors, and Associate Professors at SGUB FM.

### Section II: non-normalized performance metrics and rank correlations

As we delve into the comprehensive analysis of the SGUB FM promotion matrix, Section II focuses on the non-normalized performance metrics across the faculty ranks. Here, we explore the intrinsic values of each pillar's metrics, investigating their relationship with the faculty rank without the influence of z-score normalization. This approach allows us to observe the pure distributions and identify the particular metrics that delineate the normative institutional values inherent to each faculty rank. Through non-parametric Kruskal-Wallis testing and correlation analyses, we aim to discern the patterns and associations that might define the promotion trajectory within our institution. This section, therefore, offers an unfiltered lens through which we can understand the contributions and achievements that distinguish instructors, assistant professors, and associate professors at SGUB FM.

**Figure 5.**
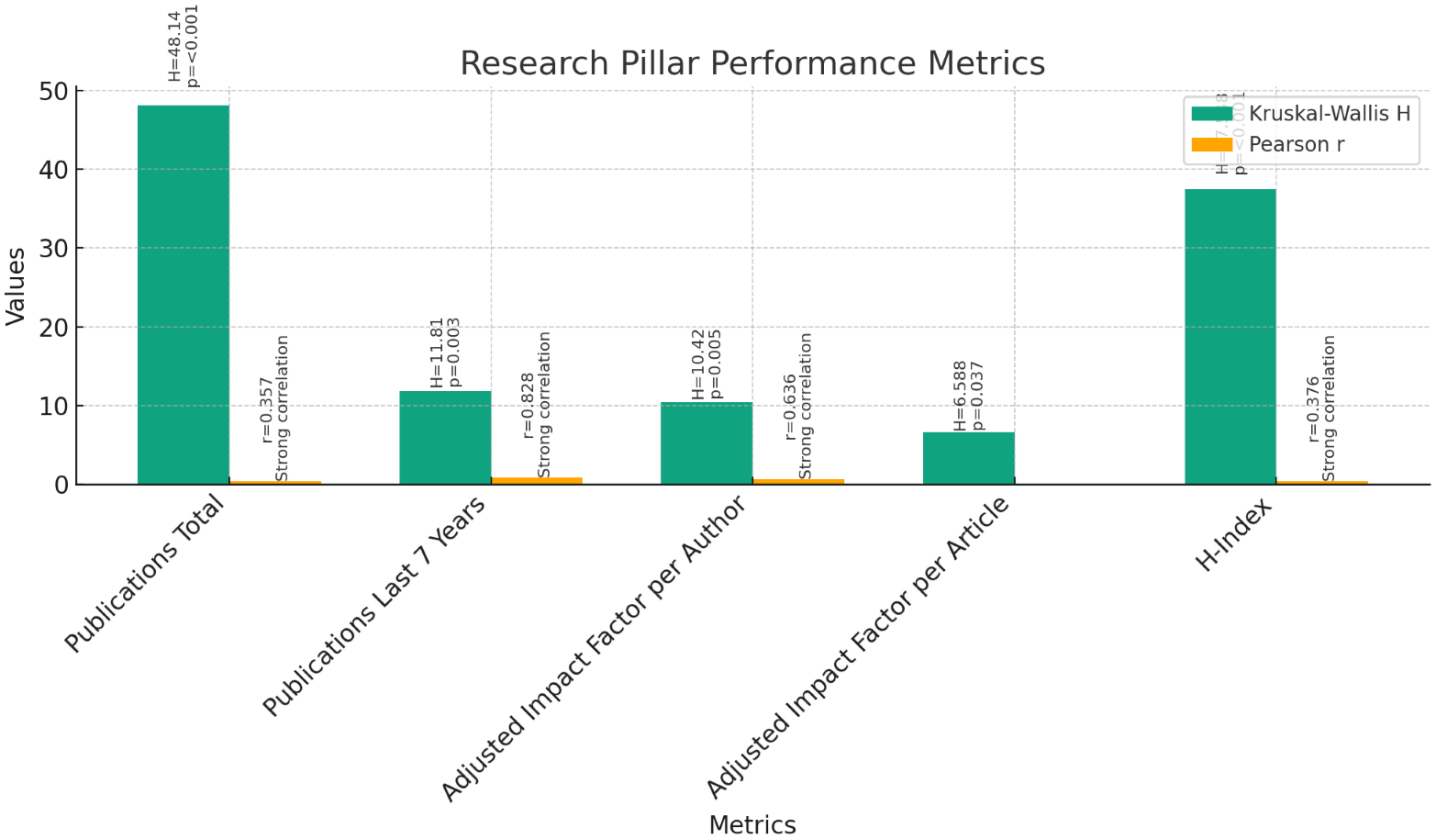
Research Pillar Performance Metrics.

**Figure 6.**
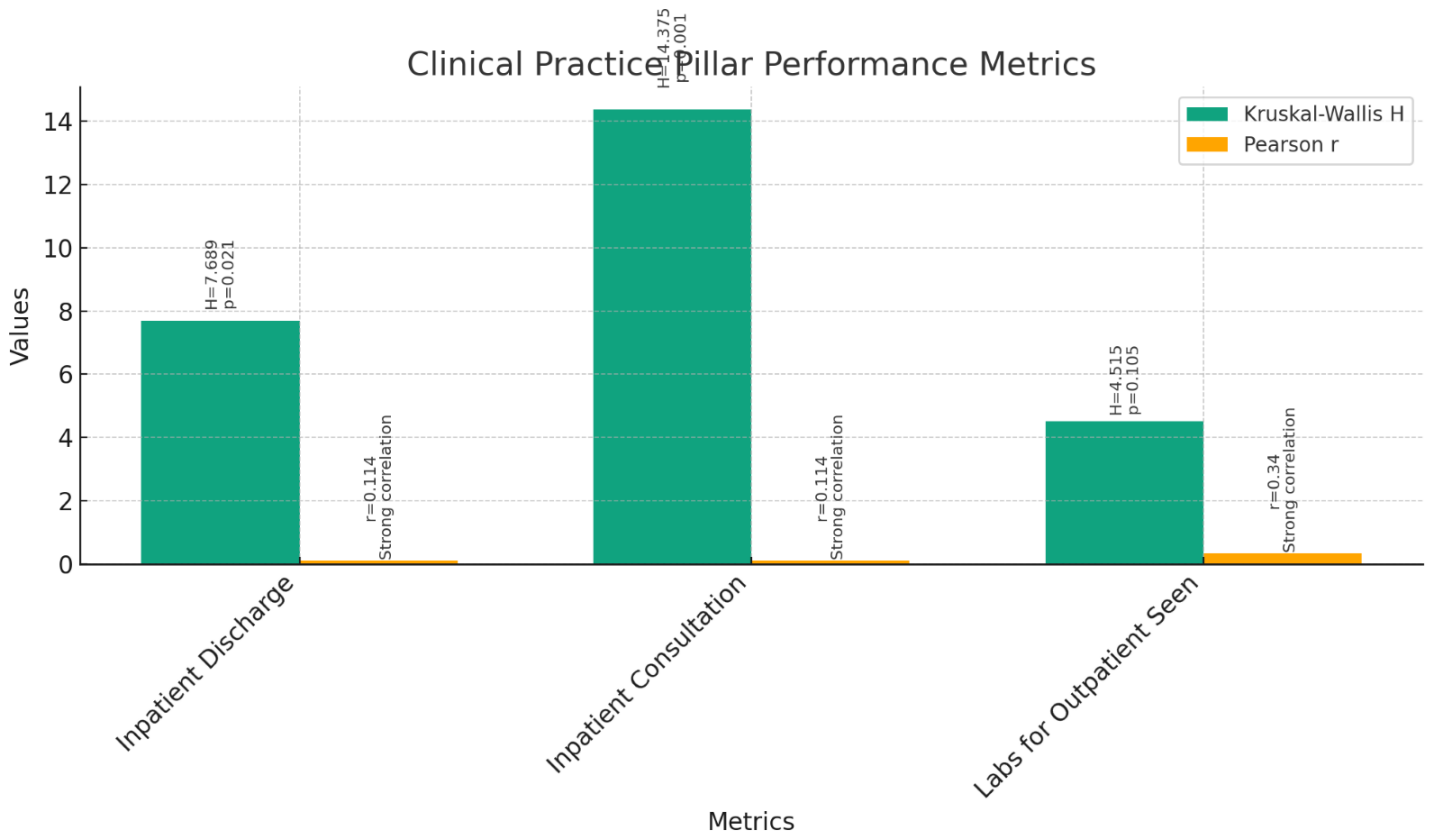
Clinical Practice Pillar Performance Metrics.

**Figure 7.**
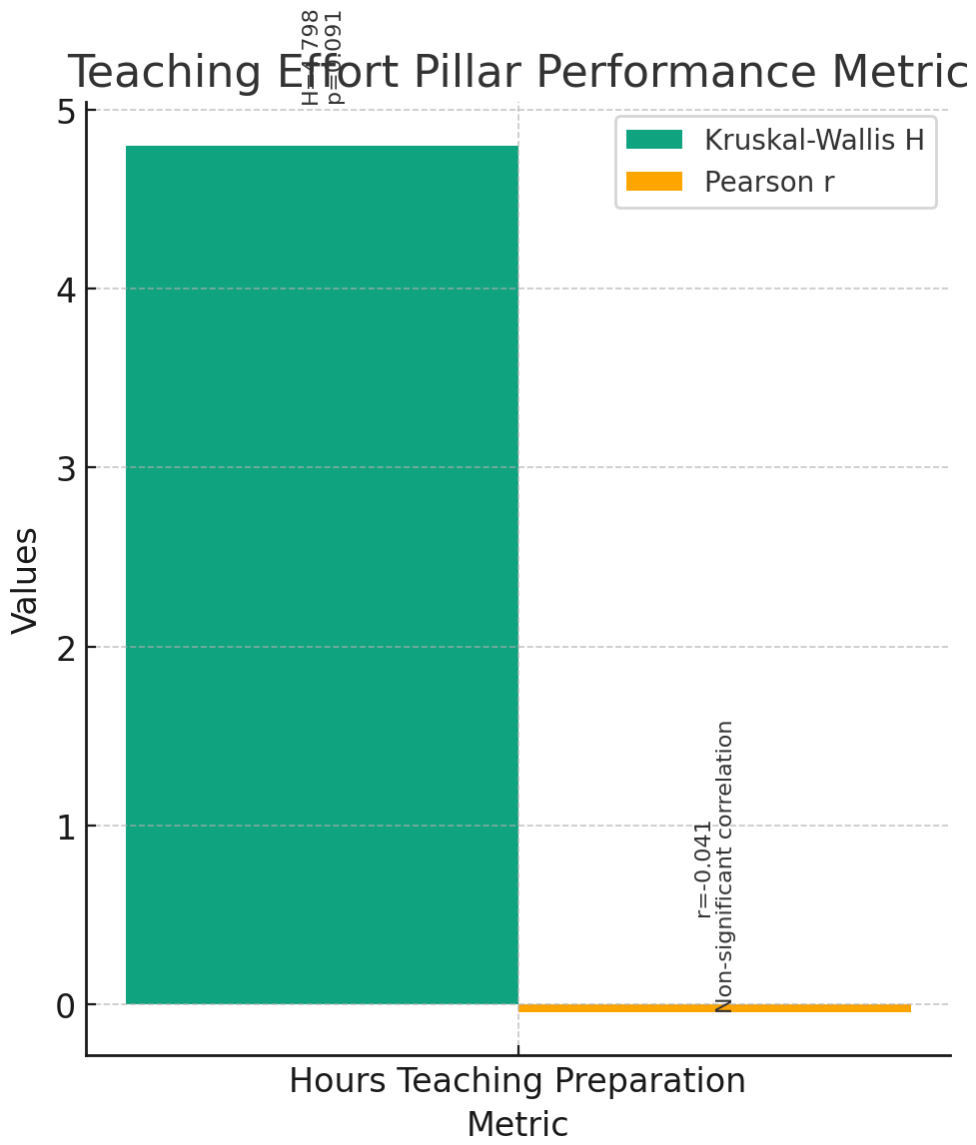
Teaching Effort Pillar Performance Metric.

**Figure 8.**
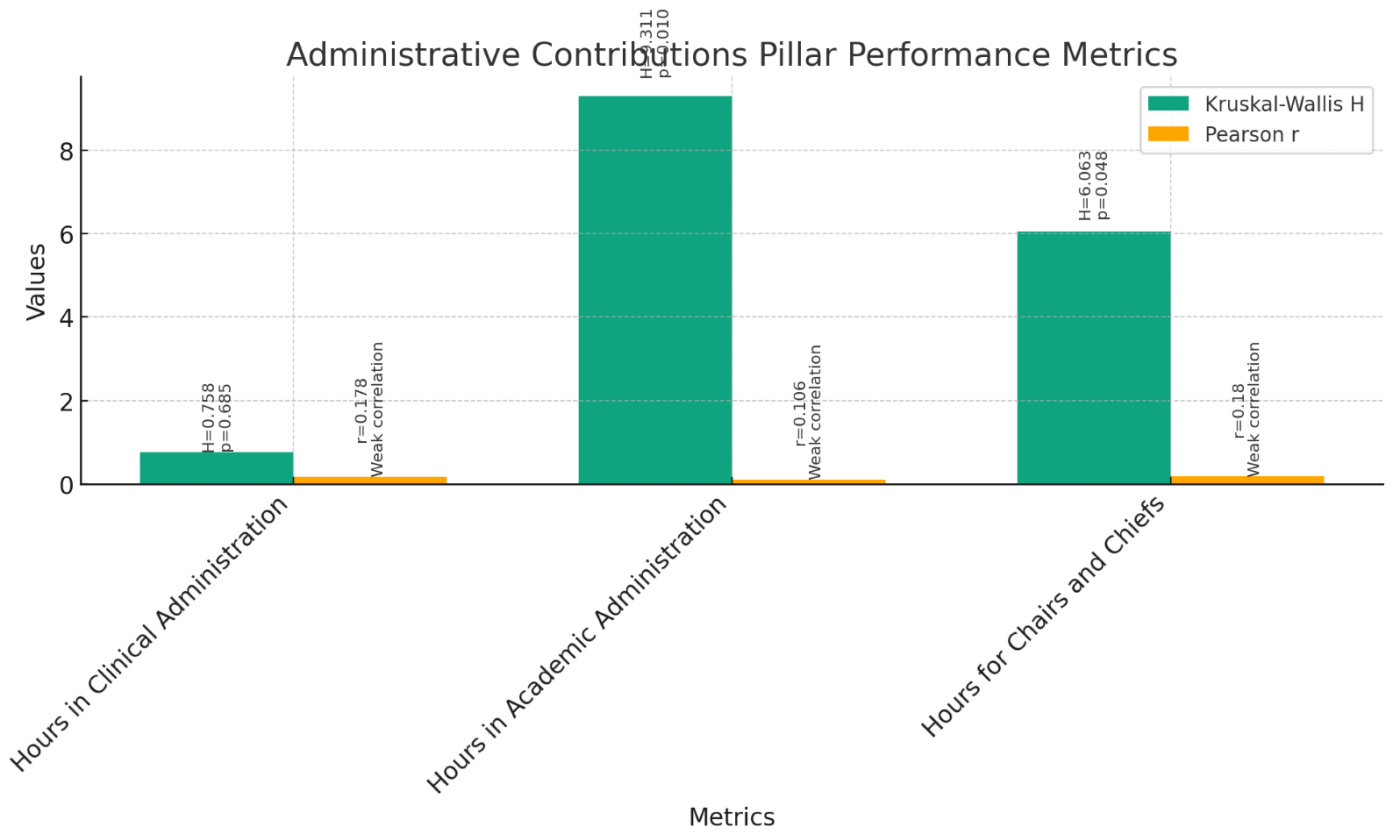
Administrative Contributions Pillar Performance Metrics.

**Figure 9.**
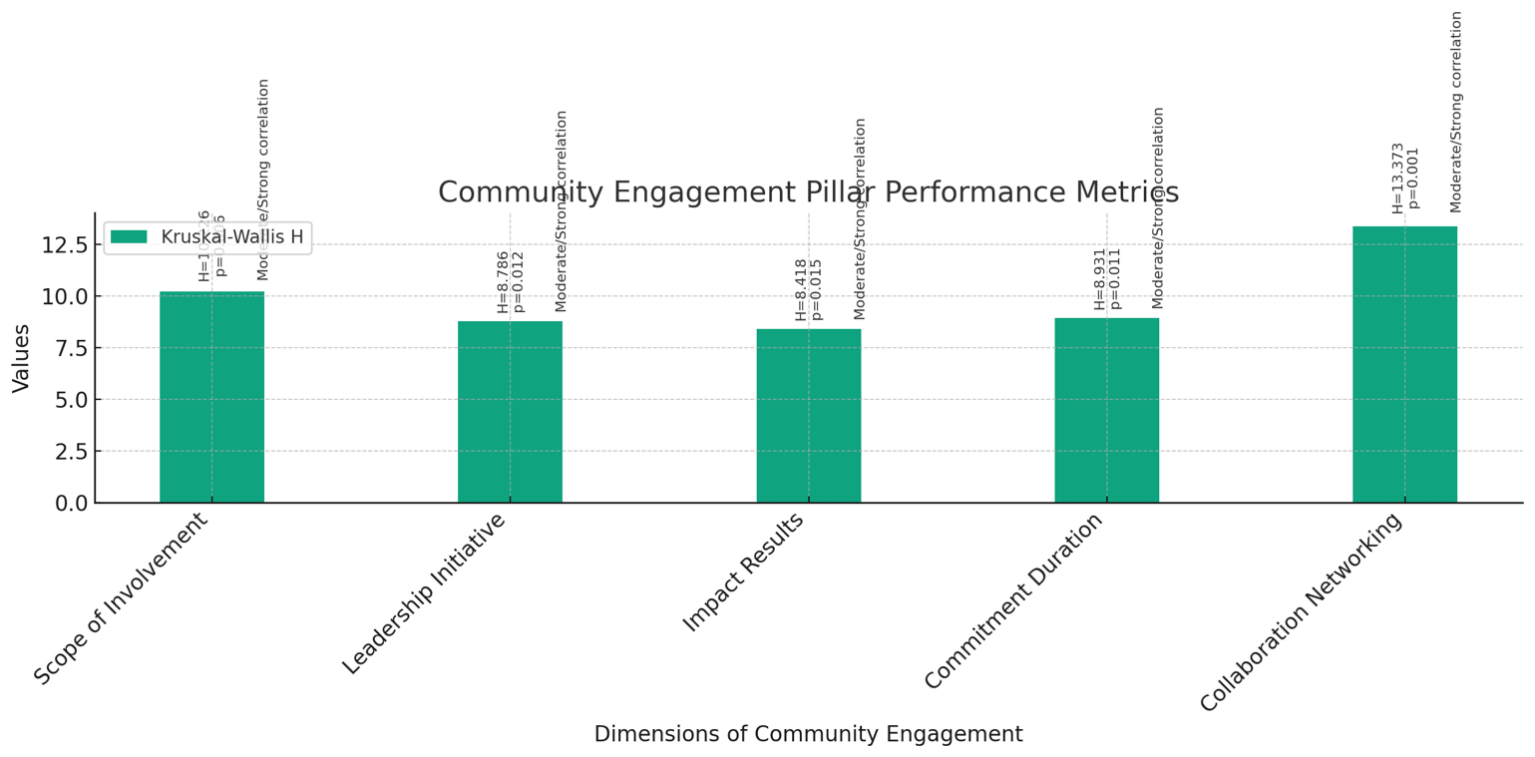
Community Engagement Pillar Performance Metrics.

**Figure 10.**
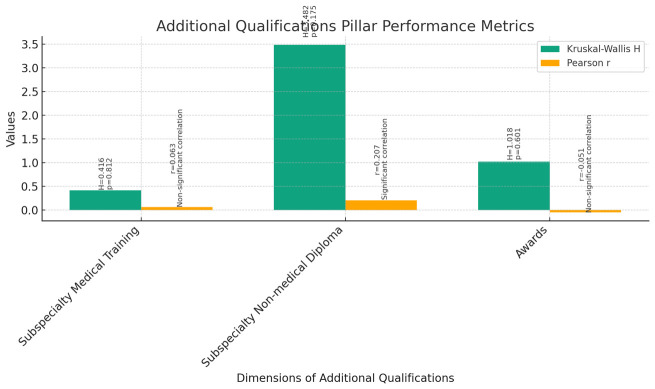
Additional Qualifications Pillar Performance Metrics.

1 **Research Pillar ( Figure 5):** The Kruskal-Wallis test was conducted to compare the performance of instructors, assistant professors, and associate professors across the various metrics of the research pillar. The results indicated significant differences among the three groups for all metrics: Publications Total (H = 48.140, p < 0.001), Publications in the Last 7 Years (H = 11.810, p = 0.003), Adjusted Impact Factor per Author (H = 10.420, p = 0.005), Adjusted Impact Factor per Article (H = 6.588, p = 0.037), and H-Index (H = 37.548, p < 0.001).
  Further analysis through pairwise comparisons revealed that associate professors had significantly higher ranks in all metrics compared to instructors and assistant professors. Assistant professors also demonstrated higher ranks than instructors across all metrics, although the differences were less pronounced.
  Correlation analyses were conducted to explore the relationship between current rank and research productivity metrics. Pearson correlations showed significant positive correlations between current rank and Publications Total (r = 0.357, p < 0.01), Publications in the Last 7 Years (r = 0.828, p < 0.01), Adjusted Impact Factor per Author (r = 0.636, p < 0.01), and H-Index (r = 0.376, p < 0.01). Spearman's rho correlations confirmed these findings, indicating strong positive correlations between current rank and research productivity metrics (p < 0.01).
  These findings suggest that as faculty members progress in rank, their research productivity tends to increase. Associate professors, in particular, demonstrate higher levels of research output compared to instructors and assistant professors.
2 **Clinical Practice Pillar ( Figure 6):** The Kruskal-Wallis test was conducted to compare the performance of instructors, assistant professors, and associate professors across various metrics within the Clinical Practice pillar. The results indicated significant differences among the three groups for Inpatient Discharge (H = 7.689, p = 0.021), Inpatient Consultation (H = 14.375, p = 0.001), and Labs for Outpatient Seen (H = 4.515, p = 0.105).
  Further analysis through pairwise comparisons revealed that associate professors had significantly higher ranks in Inpatient Discharge and Inpatient Consultation compared to instructors and assistant professors. However, there were no significant differences between the groups for Labs for Outpatient Seen.
  Correlation analyses were conducted to explore the relationship between current rank and clinical practice metrics. Pearson correlations showed significant positive correlations between current rank and Inpatient Discharge (r = 0.114, p < 0.05), Inpatient Consultation (r = 0.114, p < 0.05), and Labs for Outpatient Seen (r = 0.340, p < 0.01). Spearman's rho correlations confirmed these findings, indicating strong positive correlations between current rank and clinical practice metrics (p < 0.01).
  These findings suggest that as faculty members progress in rank, their involvement in clinical practice tends to increase, particularly in terms of inpatient activities. However, there is no significant difference in outpatient activities between different ranks.
3 **Teaching Effort ( Figure 7):** The Kruskal-Wallis test was conducted to compare the performance of instructors, assistant professors, and associate professors across the metric of Hours Teaching Preparation at SGUB. The results indicated no significant differences among the three groups (H = 4.798, p = 0.091).
  Correlation analyses were conducted to explore the relationship between current rank and the metric of Hours Teaching Preparation at SGUB. Pearson correlation showed a non-significant negative correlation between current rank and Hours Teaching Preparation (r = -0.041, p = 0.666). Spearman's rho correlation also indicated a non-significant correlation (r = 0.064, p = 0.506).
  These findings suggest that there is no significant difference in the amount of time spent on teaching preparation among instructors, assistant professors, and associate professors. Additionally, there is no significant correlation between the current rank and Hours Teaching Preparation at SGUB.
4 **Administrative Contributions ( Figure 8):** The Kruskal-Wallis test was conducted to compare the performance of instructors, assistant professors, and associate professors across various teaching administrative activities, including Hours in Clinical Administration, Hours in Academic Administration, and Hours for Chairs and Chiefs. The results indicated no significant differences among the three groups for Hours in Clinical Administration (H = 0.758, p = 0.685). However, significant differences were found for Hours in Academic Administration (H = 9.311, p = 0.010) and Hours for Chairs and Chiefs (H = 6.063, p = 0.048).
  Further examination through pairwise comparisons revealed no significant differences between instructors, assistant professors, and associate professors for Hours in Clinical Administration. However, significant differences were observed for Hours in Academic Administration and Hours for Chairs and Chiefs.
  Correlation analyses were conducted to explore the relationship between current rank and teaching administrative activities. Pearson correlations showed weak positive correlations between current rank and Hours in Clinical Administration (r = 0.178, p = 0.060), Hours in Academic Administration (r = 0.106, p = 0.267), and Hours for Chairs and Chiefs (r = 0.180, p = 0.058). Spearman's rho correlations confirmed these findings, indicating weak positive correlations between current rank and teaching administrative activities.
  These findings suggest that while there are no significant differences in Hours in Clinical Administration among instructors, assistant professors, and associate professors, there are significant differences in Hours in Academic Administration and Hours for Chairs and Chiefs. Additionally, current rank shows weak positive correlations with these teaching administrative activities, implying a slight increase in involvement as faculty members progress in rank.
5. **Community Engagement ( Figure 9):** The Kruskal-Wallis test was performed to assess the differences in community engagement activities among instructors, assistant professors, and associate professors across various dimensions, including Scope of Involvement, Leadership Initiative, Impact Results, Commitment Duration, and Collaboration Networking.
  The results revealed statistically significant differences among the three groups for all dimensions: Scope of Involvement (H = 10.226, p = 0.006), Leadership Initiative (H = 8.786, p = 0.012), Impact Results (H = 8.418, p = 0.015), Commitment Duration (H = 8.931, p = 0.011), and Collaboration Networking (H = 13.373, p = 0.001).
  Correlation analyses were conducted to explore the relationship between current rank and community engagement activities. Pearson correlations showed moderate to strong positive correlations between current rank and all dimensions of community engagement, including Scope of Involvement, Leadership Initiative, Impact Results, Commitment Duration, and Collaboration Networking. Similarly, Spearman's rho correlations confirmed these findings, indicating moderate to strong positive correlations between current rank and community engagement activities.
  These findings suggest that as faculty members progress in rank from instructors to associate professors, their involvement, leadership, impact, commitment, and collaboration in community engagement activities tend to increase significantly.
6 **Additional Qualifications ( Figure 10):** The Kruskal-Wallis test was conducted to assess the differences in additional qualifications among instructors, assistant professors, and associate professors across various dimensions, including Subspecialty Medical Training, Subspecialty Non-medical Diploma, and Awards.
  The results indicated no statistically significant differences among the three groups for Subspecialty Medical Training (H = 0.416, p = 0.812), Subspecialty Non-medical Diploma (H = 3.482, p = 0.175), and Awards (H = 1.018, p = 0.601).
  Correlation analyses were performed to examine the relationship between current rank and additional qualifications. Pearson correlations revealed a significant positive correlation between current rank and Subspecialty Non-medical Diploma (r = 0.207, p = 0.028), indicating that as faculty members progress in rank, they tend to obtain more non-medical diplomas. However, there were no significant correlations found between current rank and Subspecialty Medical Training (r = 0.063, p = 0.507) or Awards (r = -0.051, p = 0.594).
  Spearman's rho correlations confirmed these findings, showing a significant positive correlation between current rank and Subspecialty Non-medical Diploma (rho = 0.196, p = 0.039), but no significant correlations were observed for Subspecialty Medical Training (rho = 0.055, p = 0.566) or Awards (rho = -0.095, p = 0.318).
  These results suggest that while there may be some association between current rank and the acquisition of non-medical diplomas, there are no significant differences in Subspecialty Medical Training or Awards among instructors, assistant professors, and associate professors.

To add robustness and validity to the cutoff scores used for academic promotions, it was essential to investigate the statistical differences within the same academic ranks between those who were promoted and those who were not. This approach aimed to identify and quantify the factors that distinctly contribute to successful promotions, thereby providing a more objective and transparent basis for setting these crucial benchmarks. Hence, the Kruskal-Wallis tests were employed across various academic ranks (instructors, assistant professors, and associate professors) to explore significant differences in a comprehensive set of metrics between promoted and non-promoted individuals.

The Kruskal-Wallis tests conducted across various academic ranks (instructors, assistant professors, and associate professors) revealed significant differences between promoted and non-promoted individuals within the same rank across a range of metrics. Instructors who received promotions demonstrated significant disparities in total publications (H = 13.008, p < 0.001), publications within the past seven years (H = 11.001, p = 0.001), adjusted impact factors per author (H = 8.040, p = 0.005), and per article (H = 6.340, p = 0.012). Further differences were noted in the H-Index (H = 3.669, p = 0.055), clinical practice (H = 5.504, p = 0.019), and hours dedicated to teaching preparation (H = 7.954, p = 0.005) and clinical administration (H = 4.982, p = 0.026). Leadership involvement (H = 9.873, p = 0.002), commitment duration (H = 8.710, p = 0.003), and collaboration and networking (H = 4.372, p = 0.037) also varied significantly among promoted instructors.

Similarly, assistant professors who received promotions exhibited significant disparities in total publications (H = 8.212, p = 0.004), adjusted impact factors per author (H = 4.871, p = 0.027), per article (H = 8.381, p = 0.004), and mean Z-scores for research (H = 8.735, p = 0.003). Variations were also observed in hours dedicated to teaching preparation (H = 11.832, p = 0.001), clinical administration (H = 7.108, p = 0.008), and academic administration (H = 9.313, p = 0.002). Moreover, differences in involvement in leadership initiatives (H = 13.310, p < 0.001), commitment duration (H = 6.071, p = 0.014), and collaboration and networking (H = 8.041, p = 0.005) were significant among promoted assistant professors.

For associate professors, significant differences were observed between those who were promoted and those who were not in total publications (H = 6.686, p = 0.010), publications within the past seven years (H = 9.075, p = 0.003), and mean Z-scores for research (H = 9.460, p = 0.002). Significant disparities also emerged in academic administration hours (H = 10.788, p = 0.001) and involvement in leadership initiatives (H = 12.349, p < 0.001), alongside commitment duration (H = 7.730, p = 0.005) and collaboration and networking (H = 3.031, p = 0.051).

In conclusion, our comprehensive analysis of the SGUB FM promotion matrix through the Kruskal-Wallis test and correlation techniques has revealed that promotions within our academic institution are influenced by a diverse set of factors, encompassing research output, impact factors, professional engagement in teaching, clinical practice, administration, and leadership roles. The complexity and breadth of these factors highlight the multifaceted nature of academic advancement, emphasizing that successful promotion relies not only on scholarly contributions but also on effective involvement in broader institutional responsibilities. Specifically, while associate professors often exhibit superior performance in research and clinical responsibilities, no substantial differences in teaching preparation time were found across ranks. Distinct variations in administrative contributions, particularly in academic administration and leadership roles, suggest a modest yet progressive increase in administrative engagement with advancing rank. Additionally, a noteworthy finding showed that community engagement intensifies with faculty progression, underlining the increasing involvement in broader academic and social initiatives. Conversely, additional qualifications such as non-medical diplomas displayed some rank-based associations, but medical training and awards did not demonstrate significant variability among ranks. This section underscores that as faculty members ascend the academic ladder at SGUB FM, their contributions diversify and intensify, reflective of the institution's commitment to fostering professional growth and recognizing multifaceted excellence.

## Discussion

The promotion process outlined in this study involves evaluating six pillars separately, with z-scores calculated to standardize performance metrics within each. These z-scores provide a comparative measure of each faculty member's performance against their peers in the same aspect. However, when combining these z-scores for an overall weighted score, it's crucial to recognize that each pillar may represent different facets of a faculty member's contribution, such as research productivity, clinical practice, teaching effort, administrative involvement, community work, and additional qualifications or awards. As such, the final score should be seen as a composite measure reflecting the faculty member's overall performance across multiple dimensions.

Our analysis, particularly in Section II, supports this composite approach by showing that as faculty members ascend in rank, their contributions and involvements tend to broaden and intensify, demonstrating a progressive increase in various areas crucial to the institution's fabric.

It's important to note that this composite score doesn't directly compare individual z-scores across different pillars. For instance, a high z-score in research productivity doesn't necessarily mean high performance in teaching or clinical practice. Each pillar represents a unique aspect of their work, and the final score considers the relative importance of each aspect as determined by the assigned weights.

Moreover, descriptive statistics were applied to our dataset, indicating that all variables do not have a normal distribution. Therefore, the use of ANOVA statistics for comparison purposes between groups of instructors, assistant professors, and associate professors was not feasible. Consequently, we relied on non-parametric testing (Kruskal-Wallis H K independent samples) to perform the comparison of the results in each of the pillars between the three groups. This empirical approach has elucidated clear patterns within our non-normalized metrics that resonate with the normative values our institution holds, affirming that progression through the academic ranks at SGUB FM is accompanied by a significant enhancement in diverse dimensions of academic practice.

The promotion matrix at Saint George University of Beirut Faculty of Medicine (SGUB FM) signifies an earnest endeavor to establish a fair and unbiased system for faculty advancement and is demonstrably successful in its impartial treatment of gender and rank. Our analysis of a diverse cohort of 112 faculty members reveals a system that fosters equality, as evidenced by the balanced distribution of promotions. As SGUB FM stands at the cusp of a new era, it seeks to harmonize its nascent yet robust framework with the esteemed guidelines set forth by the Association of American Medical Colleges (AAMC).

The AAMC’s guidelines, while specifically designed for U.S. medical schools, offer a universal blueprint of academic excellence that SGUB FM is poised to adopt. These comprehensive criteria are integral to shaping a promotion matrix that not only evaluates but also inspires excellence in teaching, scholarly research, clinical practice, leadership, and service. Additionally, they confirm a strong ethos of professional development and a commitment to diversity, equity, and inclusion, values that SGUB FM holds in high regard.

Recent studies ( Jacobs *et al.*, 2020; Xierali *et al.*, 2021) emphasize the challenges faced by clinical educators and disparities in faculty promotion rates, underscoring the need for tailored promotion criteria and diversity promotion. Our initial criteria align with meritocratic standards, and we aim to evolve our promotion process to encompass the holistic approach advocated by the AAMC.

Our initial promotion criteria, benchmarked against venerable institutions such as AUB ( American University of Beirut, 2018), Cornell ( Cornell University, n.d.), and NYU Grossman ( New York University Grossman School of Medicine, n.d.), align with SGUB FM's meritocratic ethos. We’ve observed that our faculty, regardless of their hierarchical position, are recognized for their merits in line with objective standards similar to those practiced by our regional and international peers. The next evolution of our promotion process will encompass the holistic approach championed by the AAMC, advocating for the development of teaching portfolios that reflect the full spectrum of faculty pedagogical engagements and for evaluating the recommendations provided from peers in assessing promotion based on merit.

To facilitate this progression, SGUB FM is actively engaging in capacity-building initiatives that empower our faculty to document and highlight their achievements, particularly in the realms of teaching and research. Such initiatives echo the practices of our counterparts, preparing our faculty to meet and exceed the established benchmarks of academic medicine. We are inspired by the methodologies of AUB and NYU Grossman, which emphasize the importance of teaching portfolios and service to the profession as pivotal for promotion.

As SGUB FM enhances its promotion matrix, qualitative evaluations will add a new dimension to the promotion discourse. Peer review and evaluation processes will remain integral, evolving to ensure alignment with international best practices. We recognize the complexity of academic medicine and remain dedicated to refining evaluation processes to foster a culture of innovation and excellence.

We recognize the burgeoning complexity of academic medicine and the varied roles that faculty members have. As SGUB FM matures, it remains dedicated to refining its evaluation processes, aspiring to support a culture where innovation and distinction in medical education are the norm. This culture will foster a climate that not only acknowledges accomplishments but also catalyzes the continual professional growth of our faculty.

In embracing this multifaceted approach, we are also cognizant of the findings from Section II, which highlighted the absence of significant differences in outpatient clinical activities and teaching preparation time across ranks, suggesting that these areas of faculty work are not influenced by academic standing. Such insights compel us to ensure that our promotion matrix is not only reflective of global standards but also resonates with the intrinsic values and metrics that are pivotal for each faculty rank at SGUB FM.

In summary, SGUB FM's promotion matrix, initially tailored to suit a new institution, has now set its sights on a broader, more robust set of criteria reflective of global standards. Faculty are advised to become conversant with the forthcoming criteria, aligning their academic pursuits with the strategic vision of SGUB FM. Through this alignment, we anticipate forging an academic environment that is synonymous with excellence, innovation, and equitable opportunity for advancement.

## Ethics and consent

This study was conducted with the written approval of the Institutional Review Board (IRB) at Saint George University of Beirut Faculty of Medicine (SGUB FM). The ethical approval was granted under the reference number IRB-REC/O/021-24/1124 issued on 19 June 2024.

### Participant consent

All participants in this study provided informed consent prior to their participation. The consent process was conducted as follows:

1. Type of Consent: Written consent was obtained from all participants.
2. Consent Process: Participants were provided with a detailed information sheet outlining the study's purpose, procedures, potential risks, and benefits. They were given the opportunity to ask questions and were assured of their right to withdraw from the study at any time without any consequences.

The ethical guidelines followed in this study adhered to the principles set forth in the Declaration of Helsinki and the regulations established by the SGUB FM IRB. The anonymity and confidentiality of the participants were maintained throughout the study, and all data were securely stored and accessed only by authorized personnel.

## Data Availability

Figshare: https://doi.org/10.6084/m9.figshare.25713831 .v1 ( Jreij, 2024) This project contains the following underlying data: •   ARPP-SGUB Data are available under the terms of the Creative Commons Attribution 4.0 International license (CC-BY 4.0).
