# Implementing a multidimensional faculty promotion matrix at Saint George University of Beirut Faculty of Medicine

**DOI:** 10.12688/mep.20416.2

**Published:** 2025-04-03

**Authors:** Alexandre Nehme, Carmen El Khoury, Marc Jreij, George Karam, Ghewa El Achkar, Ziad Tannous, Nadime Cortas

**Affiliations:** 1 Faculty of Medicine, Saint George University of Beirut, Beirut, Lebanon; 2 Saint George Hospital University Medical Center, Beirut, Beirut Governorate, Lebanon

**Keywords:** Faculty Promotion, Multidimensional Evaluation, Academic Metrics, Medical Education, Institutional Development, Performance Assessment, Faculty Development Programs

## Abstract

This paper outlines the development and implementation of a multidimensional faculty promotion matrix at Saint George University of Beirut Faculty of Medicine (SGUB FM). The matrix, designed to provide a comprehensive and equitable evaluation of faculty across multiple dimensions, is anchored in six pillars: Research, Clinical Practice, Teaching Effort, Administrative Effort, Community Work, and Extra Degrees and Awards. These pillars encompass diverse components, including publication output, clinical activities, teaching responsibilities, administrative roles, community engagement, and additional qualifications, with each metric standardized using z-scores for fair comparison.

This matrix analyzed the CVs and relevant documents of 112 faculty members, demonstrating its efficacy in providing equitable evaluation regardless of gender or rank. The results showed no significant differences in promotion rates among various faculty ranks, highlighting the matrix’s fairness and impartiality. The study also explores the relationship between faculty ranks and various performance metrics, revealing patterns in research productivity, clinical practice, and community engagement that escalate with higher academic ranks.

SGUB FM's approach aligns its curricular designs and instructional implementations with international benchmarks, particularly those set by the Association of American Medical Colleges (AAMC), ensuring global standard compliance while catering to the institution's unique context. The matrix serves not only as an evaluation tool but also as a catalyst for faculty excellence and professional development. This case study offers valuable insights for medical institutions developing inclusive promotion criteria and emphasizes the importance of holistic evaluation frameworks in fostering academic excellence and professional growth.

## Introduction

Faculties of medicine worldwide encounter significant challenges in establishing comprehensive and multidimensional matrices that guide promotion processes. Historically, medical school promotions have heavily favored research productivity, prompting a reassessment of traditional metrics such as peer-reviewed papers and citations ( Dhulkhed *et al.*, 2016; Gusic *et al.*, 2014). However, a universal standard for promotions remains elusive, with institutions often developing criteria tailored to their unique needs ( Salajegheh *et al.*, 2022). There is an increasing emphasis on a holistic approach that incorporates teaching and clinical performance ( Rifai *et al.*, 2019). Teaching assessment has evolved to include multidimensional criteria that focus on fairness and comprehensive methods for faculty improvement, reflecting the faculty member’s contribution to the overall quality of education. Teaching excellence acknowledges the value of scholarly activities, a significant component to be weighed in any promotion matrix ( Glassick, 2000; Ory, 2000).

The development of robust faculty promotion frameworks is a cornerstone of academic advancement across global medical institutions. Institutions such as the Association of American Medical Colleges (AAMC) emphasize the necessity of recognizing diverse faculty contributions, including research, teaching, clinical practice, and community engagement, without prescribing specific weighting systems. This flexibility allows universities to adapt promotion frameworks to their unique missions while adhering to international standards ( AAMC, 2024). A comparative analysis of promotion systems worldwide reveals significant heterogeneity in their criteria and emphasis. U.S. medical schools, for instance, often adopt pathways for educational excellence, but less than half have well-defined criteria for teaching and leadership evaluation ( Hoffman *et al.*, 2019). Similarly, studies from the United Kingdom highlight gaps in formal teacher training and the need for comprehensive faculty development programs that integrate reflective practices and peer feedback ( Sorinola, 2014).

The absence of universal criteria poses a significant challenge for emerging medical schools, such as the Saint George University of Beirut Faculty of Medicine (SGUB FM), which lacks a foundation of established research output and prior teaching records as benchmarks for assessment. SGUB FM was founded to serve as a complementary academic entity to the Saint George Hospital University Medical Center (SGHUMC)—Lebanon’s oldest teaching medical facility, established in 1878—enhancing its academic and operational mission at all times. The faculty officially launched its academic journey by welcoming its inaugural cohort of medical students in the fall of 2022.

Despite facing the challenges of being a nascent institution, SGUB FM has embraced innovative instructional strategies aligned with its curriculum. This approach emphasizes the importance of a promotion system that recognizes the diverse contributions of its clinical faculty and supports their continuous growth and excellence ( Glassick, 2000; Ory, 2000).

The promotion matrix at SGUB FM is designed to address existing limitations in faculty evaluation by providing a multidimensional and contextually relevant framework. By integrating internationally recognized pillars—research, teaching, clinical practice, administrative roles, and community engagement—the matrix aligns closely with global best practices while introducing unique adaptations tailored to the local academic and healthcare landscape. For instance, the use of z-score standardization ensures equitable evaluation across diverse metrics, a method that has been underutilized in existing frameworks ( Steinert *et al.*, 2012). Additionally, the matrix incorporates extra degrees and awards as a distinct evaluation pillar, recognizing lifelong learning and interdisciplinary expertise. This innovative feature enhances the fairness of the framework and supports international calls for promoting professional growth beyond traditional metrics ( Simpson *et al.*, 1994).

In line with SGUB FM’s institutional goals and its foundational purpose of complementing and enhancing the operational mission of SGHUMC, the matrix places significant emphasis on metrics within the clinical pillar. These include quantifiable measures such as inpatient admissions, the number of referrals for laboratory tests, radiology exams, and consultations within SGHUMC. These metrics are particularly valuable given the diverse nature of faculty appointments, which include part-time and hybrid roles, as well as full-time faculty with privileges to admit patients or send labs to external practices. By prioritizing these measures, the matrix fosters alignment with SGHUMC’s operational objectives and supports the symbiotic relationship between the hospital and the faculty.

This study examines both the development and evaluation of the SGUB FM faculty promotion matrix, assessing its effectiveness and applicability in ensuring a structured, transparent, and data-driven approach to academic advancement. The study is divided into two phases: (1) the development of the promotion matrix, which was designed to provide a comprehensive assessment of faculty contributions across research, teaching, clinical practice, administrative roles, and community engagement, and (2) its evaluation, focusing on the matrix's ability to ensure equitable and standardized faculty assessments.

The matrix's six-pillar structure, which includes additional qualifications such as extra degrees and awards, was evaluated for its ability to distinguish high-performing faculty members and align with global academic promotion standards.

The study aims to determine whether the matrix successfully differentiates faculty based on academic and professional contributions while maintaining consistency in promotion decisions. It also investigates whether the weighting system adequately reflects faculty performance across various domains. By analyzing the initial implementation of the promotion matrix, this study offers practical insights for emerging medical institutions developing inclusive and structured faculty evaluation models that balance traditional research criteria with broader academic contributions.

## Methods

### Eligibility criteria and institutional review process for faculty promotion

To ensure consistency and fairness in the faculty promotion process, inclusion criteria were established to reflect both institutional policies and international best practices. Faculty members were required to meet the minimum time in rank thresholds within the University of Balamand (UOB) framework: instructors must have served at least two years in their current rank, assistant professors a minimum of four years, and associate professors a minimum of seven years. Only candidates who exceeded these minimum time requirements were eligible to apply for promotion, ensuring that progression reflects both experience and readiness for advancement.

Moreover, since faculty members have dual responsibilities in both clinical and teaching domains, their eligibility to apply for the promotion process was first evaluated by distinct institutional committees:

- Clinical activities were assessed by the Peer Review and Ethics Committees at SGHUMC, which rigorously evaluated quantitative clinical metrics, including infection rates, deep vein thrombosis (DVT) rates, and other critical indicators of clinical quality and ethical practice. Faculty members who did not meet these institutional standards were deemed ineligible to proceed with the promotion process.
- Teaching activities were assessed by the Curriculum Committee at SGUB FM, following an evidence-based teaching framework grounded in 360-degree evaluations. This process incorporated feedback from students and peers to ensure the effectiveness of teaching methodologies. While the qualitative evaluations were not directly factored into the promotion matrix, faculty members were required to achieve positive evaluations as a mandatory clearance step. The promotion matrix itself prioritized quantifiable teaching metrics, such as time allocated to curriculum development and course delivery, which inform the Full-Time Equivalent (FTE) framework for workload assessment and remuneration.

A total of 112 faculty members, out of 199 successfully met these criteria and applied for promotion in this study. Prior to participation, all applicants provided informed consent, following an institutional ethics review process.

### Development of the scoring matrix

The Appointment, Reappointment, and Promotion Policy (ARPP) and the associated scoring matrix at Saint George University of Beirut Faculty of Medicine (SGUB FM) were developed through a structured, iterative process that spanned over 18 months. This effort was spearheaded by the Faculty Affairs Committee, which comprises department chairs, division chiefs, and senior faculty members, and aimed to create a framework tailored to SGUB FM's unique context as a nascent institution.

The Faculty Affairs Committee has meticulously devised a comprehensive scoring matrix for academic promotions, anchored in the principles set out by the American Association of Medical Colleges (AAMC) ( AAMC, 2024; Gusic *et al.*, 2014). This matrix, a cornerstone of SGUB FM’s Appointment, Reappointment, and Promotion Policy, assesses faculty members across six essential dimensions: research, clinical practice, teaching efforts, administrative roles, community engagement, and additional qualifications, including extra degrees or awards. While the AAMC emphasizes the importance of recognizing diverse roles of medical faculty—including research, teaching, and clinical care—it does not prescribe specific weights, allowing institutions to adapt criteria to their unique missions and values ( AAMC, 2024).

At SGUB FM, the weights were determined through benchmarking against these principles and tailored to align with the institution’s mission as a nascent medical school ( Cornell University (n.d.)). The committee extensively reviewed the promotion criteria and bylaws of local, regional, and international institutions to identify practices that align with SGUB FM's mission and operational realities. This comparative analysis provided a foundation for adapting global best practices to meet the specific needs and priorities of SGUB FM.

The development process followed a systematic methodology, characterized by structured rounds of deliberation and consensus-building among faculty affairs committee stakeholders. Key steps included:

1. Initial Review and Drafting:

 - The committee began by studying promotion frameworks from peer institutions and aligning them with SGUB FM's strategic goals.

 - Draft criteria and scoring metrics were proposed, emphasizing inclusivity and balance across clinical, research, teaching, administrative, and community contributions.

2. Iterative Refinement:

 - Faculty Affairs Committee members engaged in multiple rounds of discussions to refine the criteria and their respective weights.

 - Feedback was solicited from faculty members across departments to ensure the framework addressed diverse faculty roles and priorities.

3. Consensus Agreement:

 - The final version of the ARPP and the accompanying scoring matrix was finalized through a consensus-driven process. It incorporates a balanced weighting system that reflects the institution’s commitment to excellence across all key functions of SGUB FM faculty, including clinical practice, teaching, research, administration, community engagement, and continuous education.

The promotion process at SGUB FM is deeply rooted in its historical connection to the Saint George Hospital University Medical Center (SGHUMC), Lebanon’s oldest teaching medical facility, established in 1878. Initially, faculty ranks were assigned under the auspices of the University of Balamand (UOB), which was affiliated with SGHUMC. With the establishment of SGUB FM, a strategic decision was made to retain the faculty titles conferred by UOB—such as instructor, assistant professor, associate professor, and full professor—while temporarily excluding any consideration of demotion. During its formative years, SGUB FM strategically acknowledged previously granted ranks and assessed their eligibility for promotion within its own matrix. This approach reflects a deliberate integration of historical recognition with the dynamic academic demands of SGUB FM, ensuring a seamless transition from the University of Balamand’s system while adapting to the evolving needs of the institution.

As a newly established faculty of medicine, SGUB FM also chose not to address tenure in this promotion cycle. This deliberate decision underscores its strategic focus on capacity building and fostering the development of its faculty members. By prioritizing a solid academic foundation, SGUB FM aims to cultivate a mature and well-prepared faculty body before integrating tenure into its promotion structure.

### Scoring criteria and calculation method

The scoring matrix is based on a comprehensive evaluation sheet that incorporates all metrics across the six pillars. To standardize and compare diverse metrics, a standardization employing the z-score calculation was computed for each data point ( Calandro, 2007). The z-score, or standard score, is a statistical measurement representing the number of standard deviations a data point is from the mean of a dataset. This standardization facilitates comparison across different distributions. All the clinical faculty members that applied for promotion were grouped as per their current exiting ranks yielding to three categories, the instructor category, the assistant professor category, and the associate professor category.

The formula employed for computing the z-score for each metric within each of the six evaluation pillars is *Z*=( *X*− *μ*)/ *σ*, where *Z* is the z-score, *X* is the data point, *μ* is the mean of the dataset, and *σ* is the standard deviation. Z-scores were calculated for all criteria using SPSS, based on the sample mean and standard deviation of each metric across 112 faculty members. While Z-scores typically range from -3 to +3 under the assumption of a normal distribution, the variables analyzed in this study did not follow a normal distribution. As a result, some values exceeded the typical range of Z-scores. Despite this, the use of Z-scores ensured standardization across diverse metrics, allowing for meaningful comparisons among faculty members by adjusting for differences in scale and distribution. This approach was selected to preserve the integrity of relative differences in performance, providing a robust and equitable framework for evaluating contributions across multiple dimensions.

### Pillars of the scoring matrix ( Table 1)

**Table 1.** Scoring Matrix table summary.

#	Pillar	Description	Formula
**1**	**Research Pillar**	Evaluates research output and impact using publication metrics and academic influence. Includes Total Number of Publications, Recent Publications, Adjusted Impact Factor Per Author and Article, and H-index.	Research Pillar Score = (Total Publications Z-Score×0.20) + (RP Z-Score×0.10) + (AIFAP Z-Score×0.25) + (AIFA Z- Score×0.25) + (H-index Z-Score×0.20)
**2**	**Clinical Practice** ** Pillar (CP)**	Measures clinical performance at SGHUMC using metrics like Number of Inpatient Discharges, Inpatient Consultations, and Labs for Outpatients.	Clinical Practice Pillar Score = (ID2021 Z-Score×0.40) + (IC2021 Z-Score×0.20) + (OH2021 Z-Score×0.40)
**3**	**Teaching Effort ** **Pillar (TE)**	Focuses on instructional roles at SGUB FM, assessing overall teaching effectiveness.	Teaching Effort Pillar Score = TE Z-Score×1.00
**4**	**Administrative** ** Effort Pillar (AE)**	Evaluates engagement in clinical and academic administration at SGHUMC and SGUB, measuring hours spent in various administrative roles.	Administrative Effort Pillar Score = (CA Z-Score×0.35) + (AA Z-Score×0.35) + (CCA Z-Score×0.30)
**5**	**Community Work ** **Pillar (CW)**	Assesses engagement in community-oriented activities, using a rubric that evaluates the scope, leadership, impact, commitment, and collaboration in community work.	Community Work Pillar Score = (Scope of Involvement Z-Score x 0.20) + (Leadership & Initiative Z-Score x 0.20) + (Impact & Results Z-Score x 0.20) + (Commitment & Duration Z-Score x 0.20) + (Collaboration & Networking Z-Score x 0.20)
**6**	**Extra Degrees and** ** Awards Pillar**	Recognizes additional qualifications and awards, counting extra medical and non-medical degrees, and awards received.	Extra Degrees and Awards Pillar Score = (Extra Medical Degrees Z-Score×0.40) + (Non-Medical Degrees Z- Score×0.40) + (Awards Z-Score×0.20)

1. **Research Pillar**: The Research Pillar in our faculty evaluation framework quantifies and appreciates faculty members' research output and impact, primarily through publication metrics and their academic influence. It encompasses:

- Total Number of Publications - Counts the cumulative number of published works, constituting 20% of the Research Pillar’s weight.
- Recent Publications (RP) - Assesses publications from the last 7 years, contributing 10% to the pillar’s weight, highlighting recent research activity.
- Adjusted Impact Factor Per Author (AIFAP) and Per Article (AIFA) - These metrics, each comprising 25% of the pillar’s weight, evaluate the impact of an author's and individual articles' contributions. The adjustment considers citations, author count, publication year, and the journals' field-normalized impact.
- H-index (H) - Measures productivity and citation impact of scholarly works, accounting for 20% of the pillar's weight.

Data from Scopus and Web of Science are used for analysis. An appointed member from the Faculty Affairs Committee, with the support of the Medical Education Program Coordinator from the SGUB FM Dean’s Office, conducted a thorough review of the publications and their associated metrics for each faculty member to ensure accuracy and consistency in the evaluation process. The Research Pillar contributes 30% to the overall faculty score. The overall pillar score is calculated using a weighted average of the z-scores for these metrics, following this formula:

Research Pillar Score = (Total Publications Z-Score×0.20) +(RP Z-Score×0.10)+(AIFAP Z-Score×0.25)+(AIFA Z-Score×0.25)+(H-index Z-Score×0.20)

2. **Clinical Practice Pillar (CP)**: The CP pillar in our faculty evaluation framework is centered around clinical activities of the 112 faculty members cleared to apply for promotion by SGHUMC’s peer review and ethics committees. This pillar measures performance based on three main metrics as of 2021:

- ID2021: Number of Inpatient Discharges - This metric, constituting 40% of the CP pillar’s weight, tracks the number of patients discharged.
- IC2021: Number of Inpatient Consultations - Accounting for 20% of the pillar's weight, this metric assesses the number of consultations conducted with inpatients.
- OH2021: Number of Labs for Outpatients - This component represents 40% of the CP pillar's weight, focusing on the number of laboratory tests conducted for outpatients.

The data for these metrics is sourced from the Chief Medical Officer (CMO) office at SGHUMC, retrieved from the electronic medical records system used by the hospital (Oracle-based platforms). The Clinical Practice Pillar contributes 25% to the overall faculty score. The score for this pillar is calculated using a weighted average of the z-scores for each metric. The formula for this calculation, reflecting the stated percentages, is: Clinical Practice Pillar Score= (ID2021 Z-Score×0.40) +(IC2021 Z-Score×0.20)+(OH2021 Z-Score×0.40)

3. **Teaching Effort Pillar (TE):** The Teaching Effort (TE) pillar in our faculty evaluation framework assesses instructional roles at SGUB FM, beginning in 2021, with a focus on curriculum development and teaching efforts.

The data for this pillar is meticulously collected and validated through official sources:

- **Teaching Hours**: Logged hours are sourced from official course schedules and verified against departmental records to ensure accuracy.
- **Curriculum Development Contributions**: Documentation of faculty involvement in designing and updating course content is reviewed to reflect innovation and alignment with educational goals meticulisly recorded in the curriculum committee meetings detailing the weekly progress and contributions of every faculty to curriculum development.

This pillar accounts for 15% of the overall faculty score, ensuring the recognition of diverse teaching contributions. The score for the TE pillar is calculated using the TE Z-Score, which represents the standardized measure of hours taught and other instructional contributions, reflecting the comprehensive nature of teaching responsibilities. The formula is as follows:Teaching Effort Pillar Score = TE Z-Score × 1.00

4. **Administrative Effort Pillar (AE)**: The AE pillar in our faculty evaluation framework evaluates faculty members' engagement in clinical and academic administration at SGHUMC and SGUB. This pillar accounts for the time dedicated to various administrative roles and committee memberships, reflecting the scope of each faculty member's administrative involvement within the institution. The AE Pillar comprises three distinct components:

- CA: The number of hours spent in clinical administration at SGHUMC, contributing 35% to the AE pillar's weight retrieved from the CMO office.
- AA: The number of hours dedicated to academic administration at SGUB, also accounting for 35% of this pillar's weight.
- CCA: The time spent in higher administrative capacities such as chief or chairman roles at both SGHUMC and SGUB, constituting 30% of the AE pillar's weight.

Collectively, these components represent a comprehensive view of a faculty member's administrative contributions. The Administrative Effort Pillar contributes 15% to the overall faculty score. The score for the AE Pillar is calculated using a weighted average of the z-scores for each metric. The formula, incorporating the specified percentages, is as follows:

Administrative Effort Pillar Score= (CA Z-Score×0.35)+(AA Z-Score×0.35)+(CCA Z-Score×0.30)

5. **Community Work Pillar (CW)**: The CW Pillar in our faculty evaluation framework assesses faculty members' engagement in broader community-oriented activities and was self-reported by each applicant in the CV. This pillar captures involvement in non-governmental organizations (NGOs), ministerial committees, and national or international scientific committees. Evaluation within this pillar is multifaceted, encompassing both the depth and breadth of participation in these organizations.

To effectively quantify these contributions, a specialized rubric, accessible at Community Work Rubric, has been employed. This rubric segments the evaluation into five key areas, each equally weighted to contribute 20% of the total CW score:

- Scope of Involvement
- Leadership & Initiative
- Impact & Results
- Commitment & Duration
- Collaboration & Networking

Activities are scored on a scale of 1 to 5 in each area based on the above predefined criteria, such as the nature of the activity, its duration, demonstrated leadership, and impact. These scores are standardized using Z-scores to enable comparability across diverse faculty members and activity scales. The overall CW Pillar score is then calculated as a weighted average of the standardized Z-scores for the five areas, following the distribution outlined in the rubric. This pillar constitutes 5% of the overall faculty score. The formula for the CW Pillar is as follows:

Community Work Pillar Score = (Scope of Involvement Z-Score x 0.20) + (Leadership & Initiative Z-Score x 0.20) + (Impact & Results Z-Score x 0.20) + (Commitment & Duration Z-Score x 0.20) + (Collaboration & Networking Z-Score x 0.20)

6. **Extra Degrees and Awards Pillar**: The Extra Degrees and Awards Pillar in our faculty evaluation framework is designed to recognize faculty members’ achievements beyond traditional academic metrics. This pillar acknowledges their commitment to lifelong learning and interdisciplinary expertise, which enhance their capacity to innovate and contribute to the institution. The data for this pillar is self-reported through the CV and externally validated for accuracy by an appointed member from the Faculty Affairs Committee, with the support of the Medical Education Program Coordinator from the SGUB FM Dean’s Office. This pillar encompasses:

- Extra Medical Degrees after Fellowship - Each additional medical specialty training acquired post-fellowship is assigned a value of 1 and contributes 40% to the weight of this pillar.
- Non-Medical Degrees - Similar to medical degrees, each non-medical degree attained is valued at 1, also accounting for 40% of this pillar's weight.
- Awards - Each award received is assigned a value of 1, contributing 20% to the pillar's weight.

The evaluation within this pillar thus sums up the total number of extra degrees (both medical and non-medical) and awards, each metric contributing a designated percentage to the pillar's overall score. The Extra Degrees and Awards Pillar constitutes 10% of the overall faculty score. To calculate the score for this pillar, a weighted average of the z-scores for the metrics of extra degrees and awards is used. The formula, based on the specified weightings, is:

Extra Degrees and Awards Pillar Score=(Extra Medical Degrees Z-Score×0.40)+(Non-Medical Degrees Z-Score×0.40)+(Awards Z-Score×0.20)

Including the extra awards and additional degrees pillar in our promotion criteria for faculty members is crucial for recognizing their achievements, promoting diversity and innovation, enhancing institutional reputation, attracting and retaining talent, and encouraging lifelong learning. It acknowledges exceptional accomplishments, fosters a multidisciplinary approach, enhances credibility, and motivates continued professional development.

### Overall score calculation ( Table 2)

**Table 2.** Overall Score Calculation table summary.

#	Pillar	Weight in Total Score	Description
**1**	**Research Pillar**	30%	The score is calculated from publication metrics and impact, contributing 30% to the overall score.
**2**	**Clinical Practice Pillar (CP)**	25%	This pillar assesses clinical activities and contributes 25% to the total score.
**3**	**Teaching Effort Pillar (TE)**	15%	Focuses on teaching roles and effectiveness, representing 15% of the overall score.
**4**	**Administrative Effort Pillar** ** (AE)**	15%	Evaluates administrative roles within the faculty, accounting for 15% of the total score.
**5**	**Community Work Pillar (CW)**	5%	Measures community engagement and activities, making up 5% of the overall score.
**6**	**Extra Degrees and Awards** ** Pillar**	10%	Recognizes additional qualifications and achievements, contributing 10% to the total score.

The overall score for each candidate is determined by a weighted summation of the scores from each pillar, where each pillar's score is itself a weighted sum of its constituent metrics’ z-scores. The weight distribution for each pillar in the total score is as follows: Research (30%), Clinical Practice (25%), Teaching Effort (15%), Administrative Effort (15%), Community Work (5%), and Extra Degrees and Awards (10%). Given this, the formula for the overall score is:

Overall Score= (Research Pillar Score×0.30)+(Clinical Practice Pillar Score×0.25)+(Teaching Effort Pillar Score×0.15)+(Administrative Effort Pillar Score×0.15)+(Community Work Pillar Score×0.05)+(Extra Degrees and Awards Pillar Score×0.10)

This calculation ensures that the final score comprehensively reflects the candidate's performance across all the specified domains. Both Excel 365 and SPSS version 27 were used for calculations and statistics to ensure accuracy and reliability in the scoring process.

## Results and analysis

**Figure 1.**
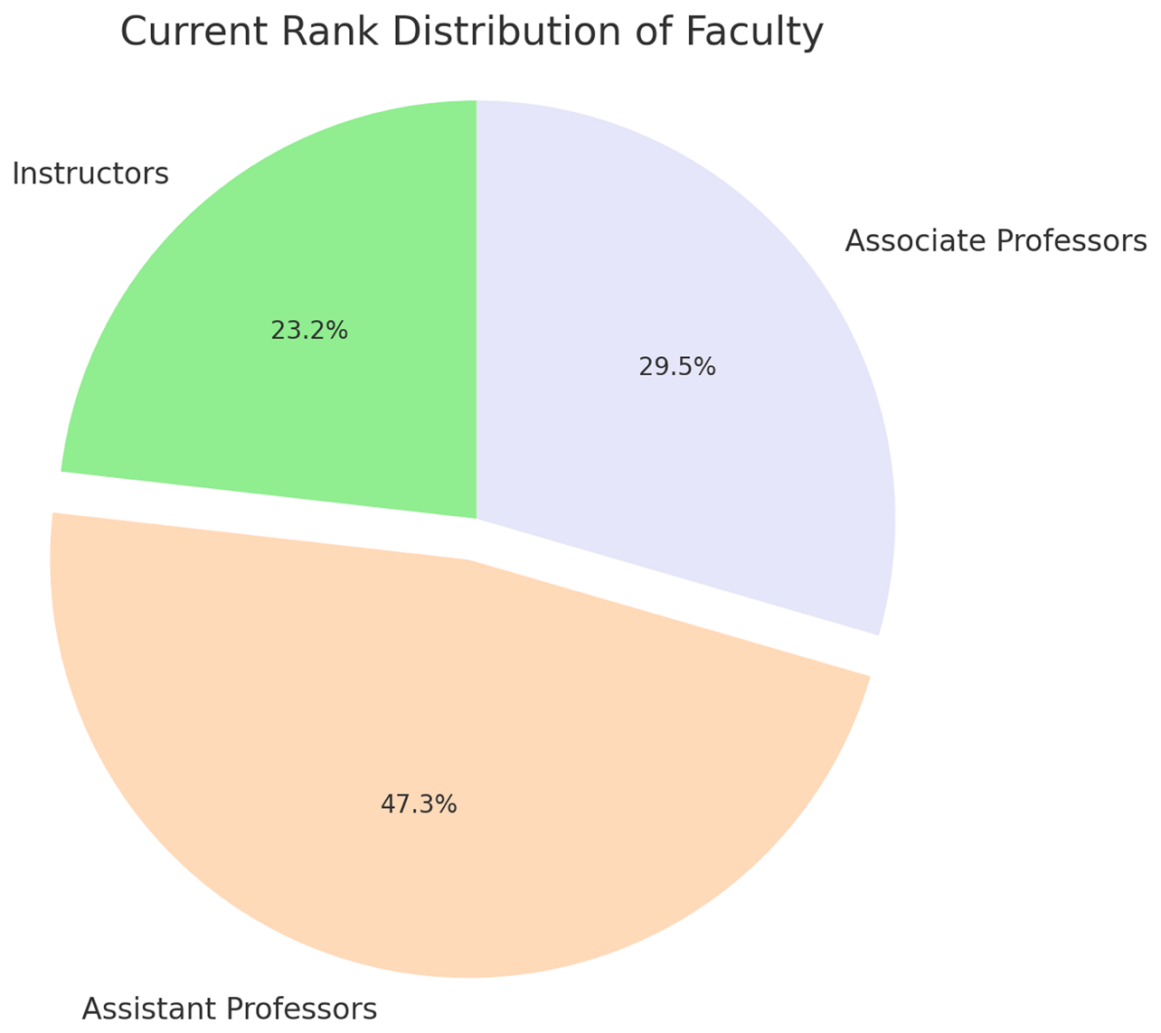
Rank Distribution of SGUB FM Faculty members.

The promotion matrix of Saint George University of Beirut Faculty of Medicine (SGUB FM) was evaluated across a dataset of 112 faculty members, representing the entire population involved in the promotion process. This ensured a comprehensive assessment with no missing entries. The total faculty population consists of 199 faculty members, including 141 males (70.9%) and 58 females (29.1%). Among those eligible for promotion, 76 were male (67.9%) and 36 were female (32.1%) ( Figure 1), closely reflecting the institution’s overall gender composition.

To statistically assess whether the gender distribution of faculty members undergoing the promotion process is representative of the entire faculty body, a Chi-Square test was conducted. The test results indicated χ²(1) = 0.379, p = 0.538, confirming no significant difference between the gender distribution of the promotion-eligible group and that of the total faculty population. This suggests that the subgroup undergoing the promotion process is statistically representative of the overall faculty population in terms of gender composition.

To evaluate gender equity in the propmotion process, statistical analyses were conducted to compare the performance of male and female faculty members across all evaluated metrics. The Kruskal-Wallis test revealed no statistically significant differences between genders in key metrics, including research (p = 0.583), teaching (p = 0.699), community engagement (p = 0.562), and overall Z-scores (p = 0.356). These findings highlight the fairness of the matrix in providing equitable evaluations across genders. Marginally non-significant trends were observed in clinical practice (p = 0.066) and administrative function scores (p = 0.054), where male faculty exhibited slightly higher mean ranks. These trends are likely reflective of historical role distributions within the faculty rather than systemic bias in the matrix itself.

The inclusion of diverse pillars—such as community work, teaching, clinical practice, and administrative contributions—ensures that the matrix recognizes a wide range of professional activities, mitigating potential biases linked to traditional metrics like research output.

The current ranks within this dataset comprise 26 instructors (23.2%), 53 assistant professors (47.3%), and 33 associate professors (29.5%) ( Figure 1).

### Section I: standardized evaluation of faculty achievement using z-scores

**Figure 2.**
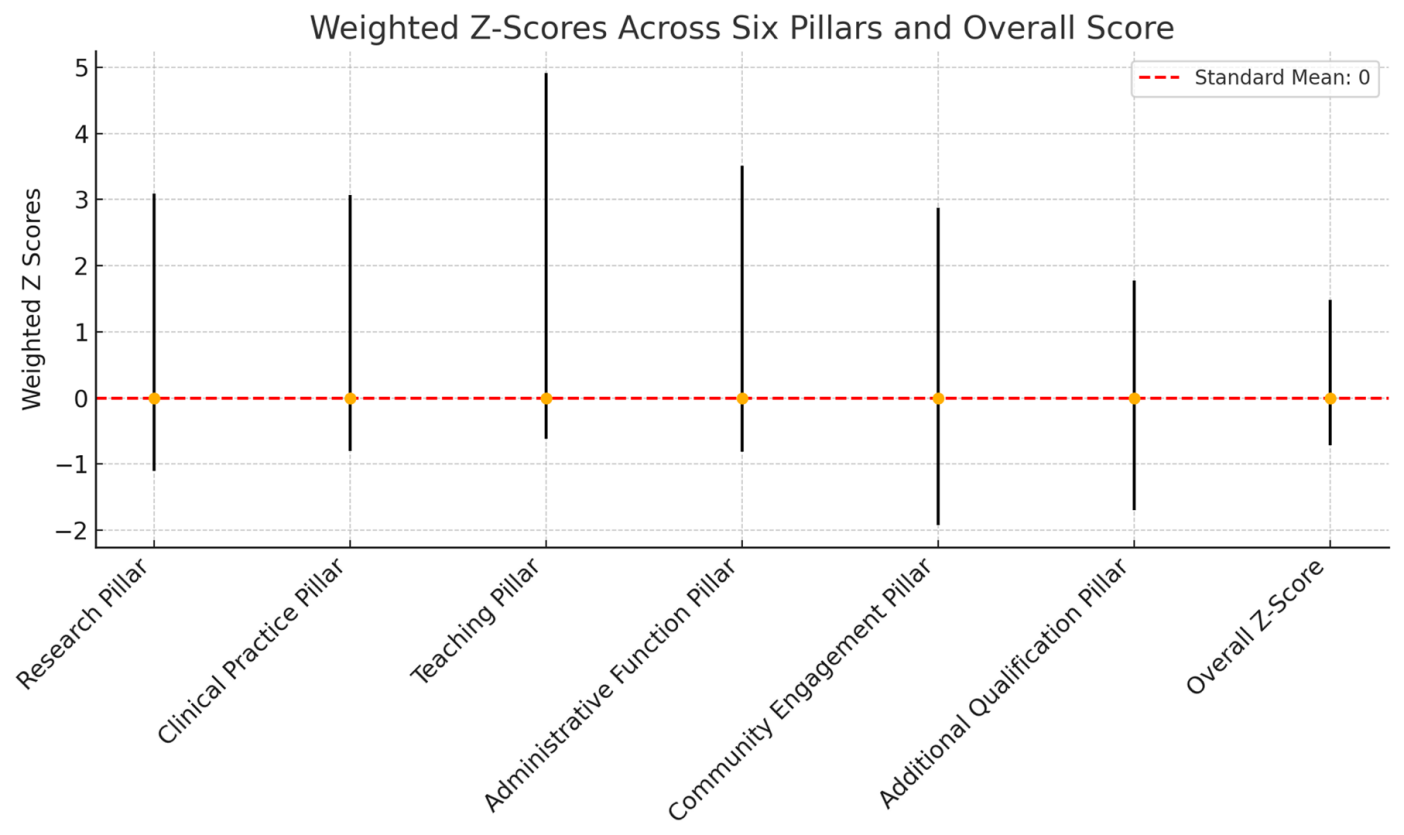
Weighted Z-score mean across the six pillars.

**Figure 3.**
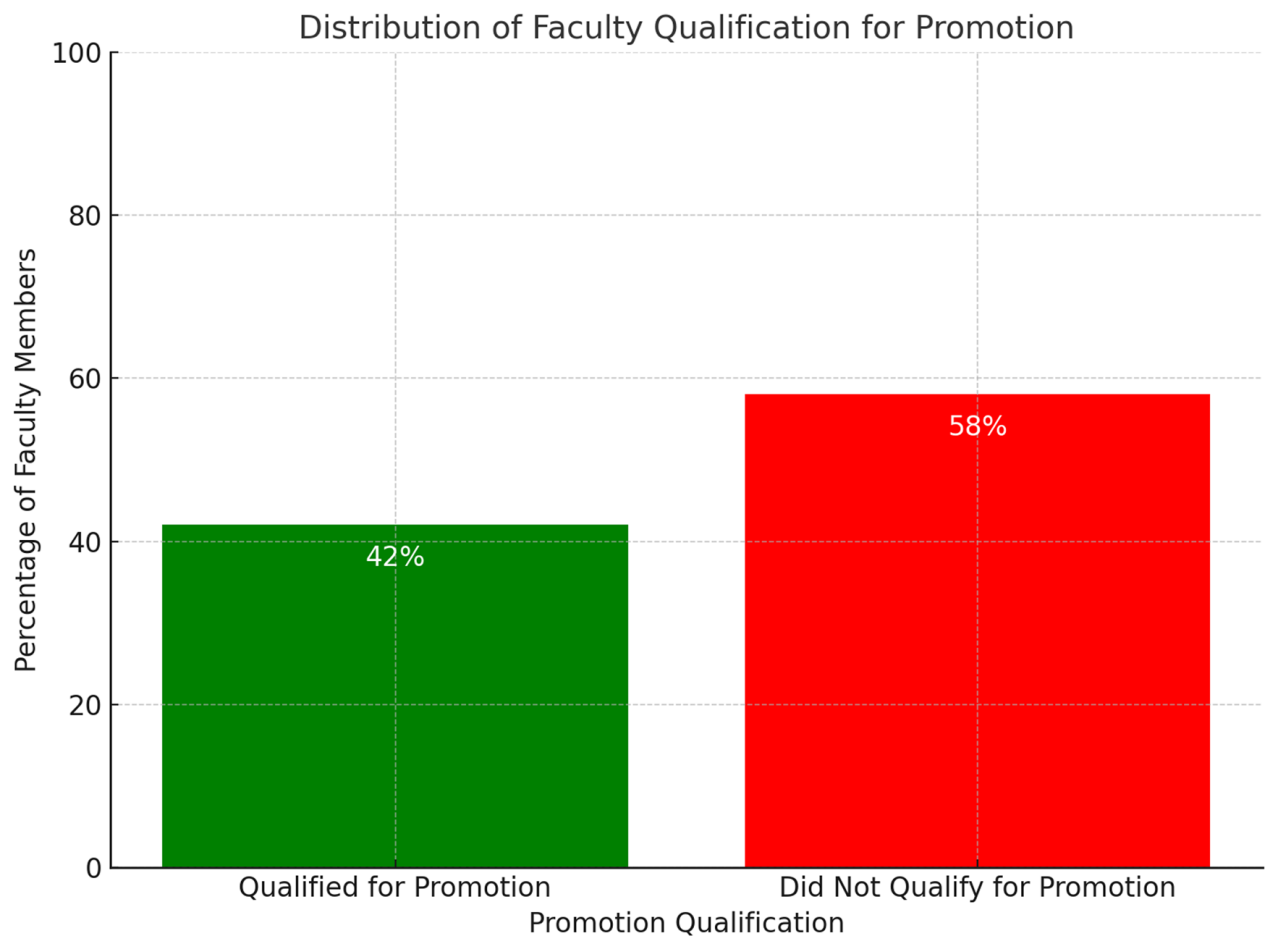
Percentage of Faculty Qualified for Promotion.

The descriptive statistics for the weighted z-scores of the six pillars reveal a consistent standardized evaluation across 112 faculty members ( Figure 2 and Figure 3).

1. **Research Pillar**: The Z-scores for Research ranged from -1.10 to 3.09, with a standardized mean of 0.00 and a standard deviation of 0.774, indicating a moderate spread around the mean ( Figure 2).
2. **Clinical Practice Pillar**: Faculty performance in Clinical Practice also centered around the standardized mean of 0.00, with Z-scores extending from -0.80 to 3.07 and a standard deviation of 0.779, suggesting comparable variability to the Research pillar ( Figure 2).
3. **Administrative Function Pillar**: For Administrative Functions, the Z-scores varied from -0.81 to 3.51, maintaining the standardized mean of 0.00. A slightly lower standard deviation of 0.720 compared to other pillars indicates a tighter clustering of scores around the mean ( Figure 2).
4. **Teaching Pillar**: The Z-scores for Teaching spanned a wider range, from -0.62 to 4.91, with a standardized mean of 0.00 and the highest standard deviation among the pillars at 0.991, reflecting greater variability in teaching contributions ( Figure 2).
5. **Community Engagement Pillar**: Community involvement displayed Z-scores ranging from -1.92 to 2.87, with a standardized mean of 0.00 and a standard deviation of 0.960, highlighting substantial diversity in community engagement activities among the faculty ( Figure 2).
6. **Additional qualifications Pillar**: The Z-scores for Additional Qualifications ranged between -1.70 and 1.77, averaging exactly at the standardized mean of 0.00 with a standard deviation of 0.679, the lowest among the pillars, indicating a more concentrated distribution of scores ( Figure 2).
**Weighted Overall Z-Score**: The aggregate of the weighted z-scores, which constitutes the Overall Z-Score, ranged from -0.72 to 1.48 with a near-standard mean of 0.00 and a standard deviation of 0.456. This suggests that when considering all pillars collectively, faculty performance is closely aligned around the mean, reflecting a balanced evaluation process ( Figure 2).

The Z-scores obtained in this study logically extended beyond the typical range of -3 to +3, as they were calculated for standardization purposes rather than normalization, and the initial data were not normally distributed, which naturally resulted in some extreme values reflecting the inherent variability in the dataset.

All results are based on the full count of valid cases (N=112), ensuring that the analysis is comprehensive and representative of the SGUB FM faculty ( Table 3). To categorize individuals into "qualified for promotion" (scores at or above 0) and "not qualified" (scores below 0), a new binary variable was created in SPSS named 'Final'. This categorization involved re coding the 'Total Score' variable such that scores below 0 were flagged as '0', indicating non-eligibility for promotion, and scores at or above 0 were flagged as '1', indicating eligibility for promotion.

**Table 3.** Metrics Z score descriptive statistics.

Variable	N	Minimum	Maximum	Mean	Std. Deviation
**WeightedZscoreResearch**	112	-1.1	3.09	0.0	0.77403
**WeightedZscoreClinical Practice**	112	-0.8	3.07	0.0	0.77917
**WeightedZscore Teaching**	112	-0.61923	4.9078	0.0	0.99095004
**WeightedZscoreAdminFunc**	112	-0.81	3.51	0.0	0.7201
**WeightedZscoreCommunity**	112	-1.92	2.87	0.0	0.9598
**WeightedZscoreExtra**	112	-1.7	1.77	0.0	0.6788
**WeightedOverallZscore**	112	-0.72	1.48	0.001	0.45626

Following the recoding process, a frequency analysis of the 'Final' variable revealed that out of 112 faculty members, 47 individuals—accounting for 42% of the faculty—achieved scores of 0 and above, thus qualifying for promotion. Meanwhile, 65 faculty members, making up 58%, scored below the 0-point threshold and did not meet the criteria for promotion.

The crosstabulation results provide an overview of the promotion outcomes across different faculty ranks at Saint George University of Beirut Faculty of Medicine. The faculty ranks, coded as 1 (Instructors), 2 (Assistant Professors), and 3 (Associate Professors), were cross-tabulated against a binary promotion outcome where '1.00' indicates those who qualified for promotion and '.00' indicates those who did not.

**Figure 4.**
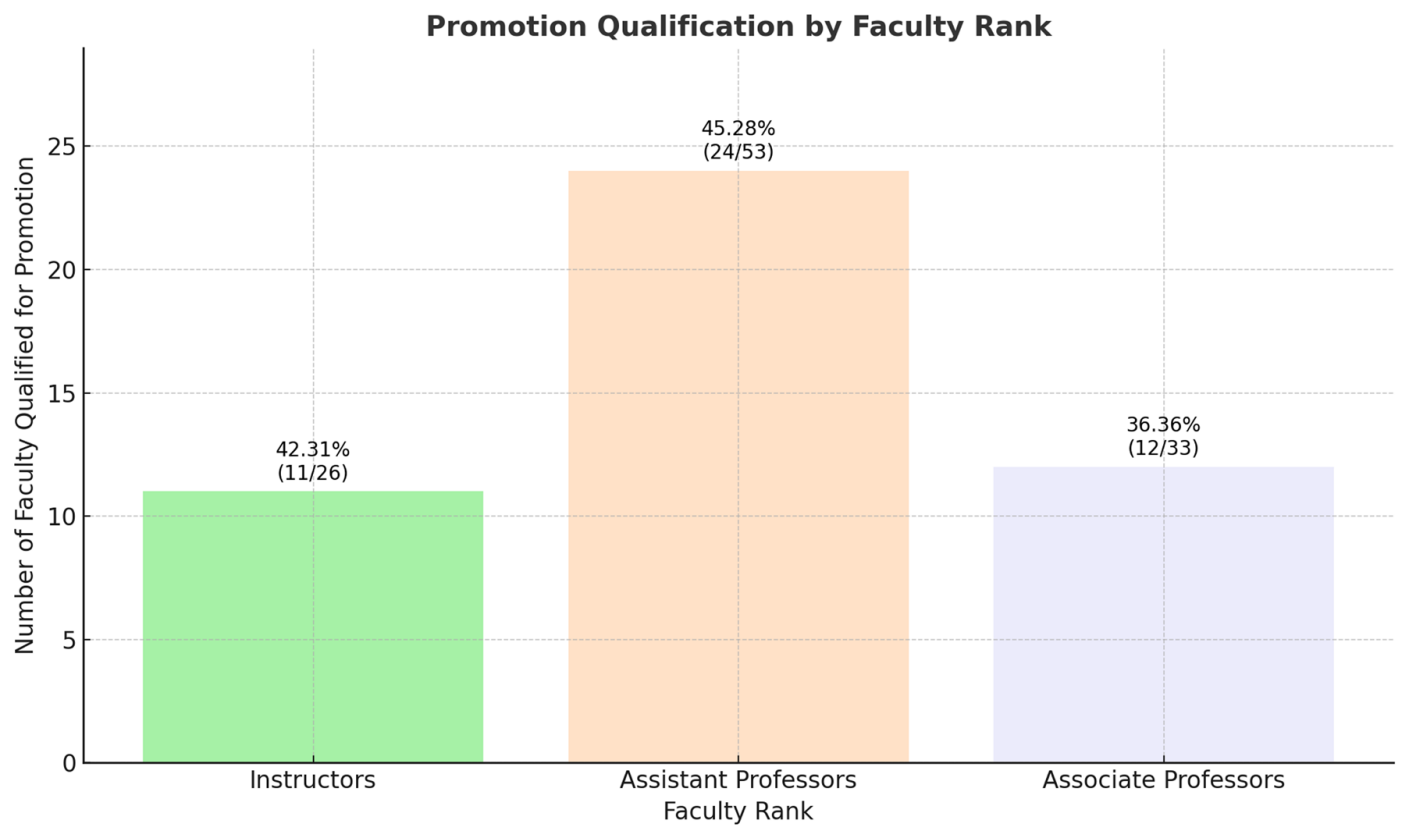
Percentage of Faculty Qualified for Promotion by rank.

The data indicates that the proportion of faculty meeting the criteria for promotion includes ( Figure 4):

- 11 out of 26 Instructors, representing roughly 42.31%,
- 24 out of 53 Assistant Professors, amounting to approximately 45.28%,
- 12 out of 33 Associate Professors, or about 36.36%.

However, the Chi-Square test of independence, with a Pearson Chi-Square value of 0.666 and a significance level (p-value) of 0.717, suggests that there is no statistically significant association between faculty rank and the likelihood of promotion. This is further supported by the Likelihood Ratio (p-value of 0.717) and the Linear-by-Linear Association (p-value of 0.610), both confirming the absence of a statistically significant trend across the ranks.

The number of valid cases was 112, and no expected cell counts were below 5, which satisfies one of the assumptions for the validity of the Chi-Square test.

In summary, the promotion process, when analyzed statistically, appears to be fair and unbiased across different faculty ranks, as there is no significant difference in promotion rates among Instructors, Assistant Professors, and Associate Professors at SGUB FM.

### Section II: non-standardized performance metrics and rank correlations

As we delve into the comprehensive analysis of the SGUB FM promotion matrix, Section II focuses on the non-standardized performance metrics across the faculty ranks. Here, we explore the intrinsic values of each pillar's metrics, investigating their relationship with the faculty rank without the influence of z-score standardization. This approach allows us to observe the pure distributions and identify the particular metrics that delineate the standardized institutional values inherent to each faculty rank. Through non-parametric Kruskal-Wallis testing and correlation analyses, we aim to discern the patterns and associations that might define the promotion trajectory within our institution. This section, therefore, offers an unfiltered lens through which we can understand the contributions and achievements that distinguish instructors, assistant professors, and associate professors at SGUB FM.

**Figure 5.**
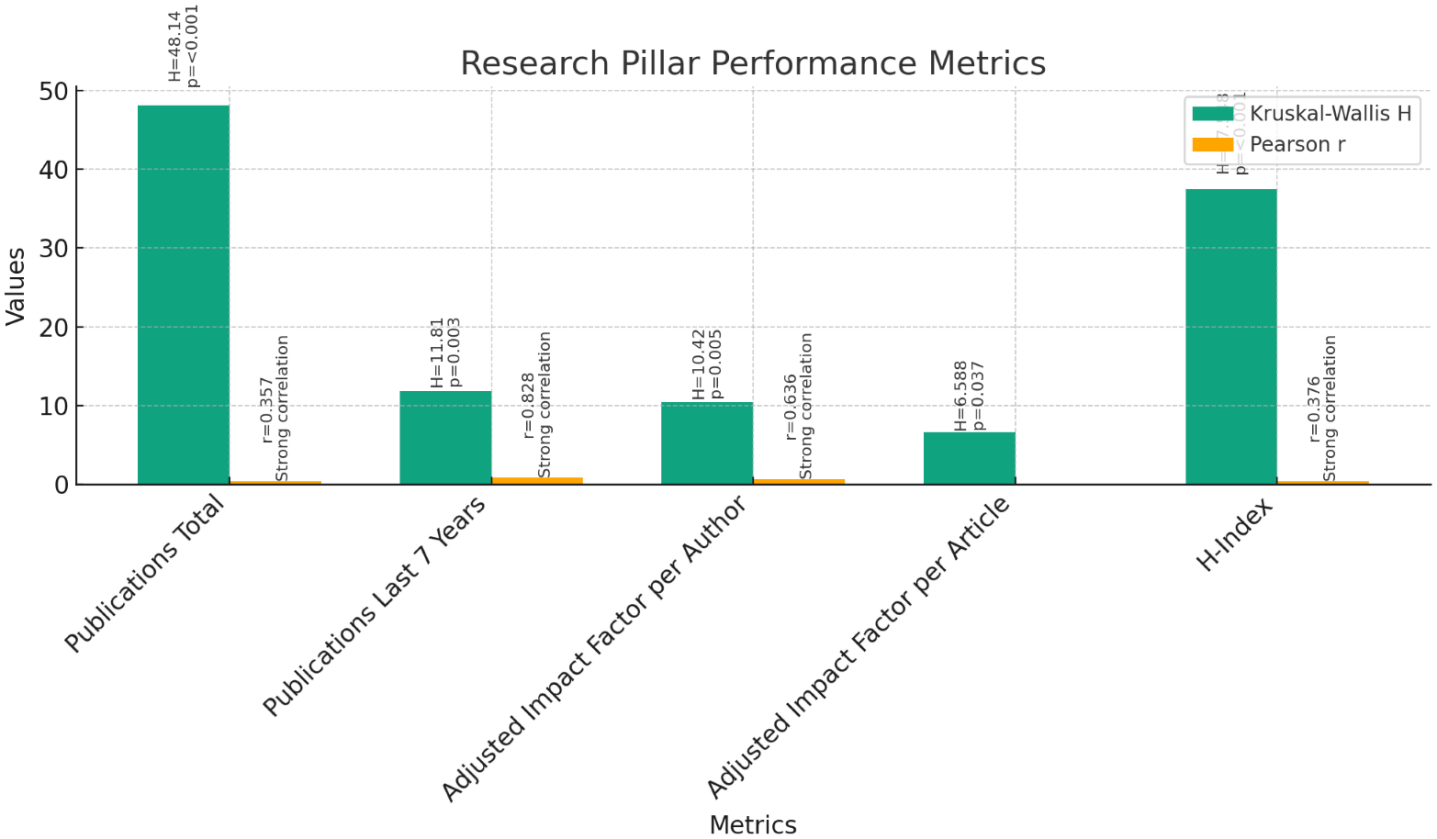
Research Pillar Performance Metrics.

**Figure 6.**
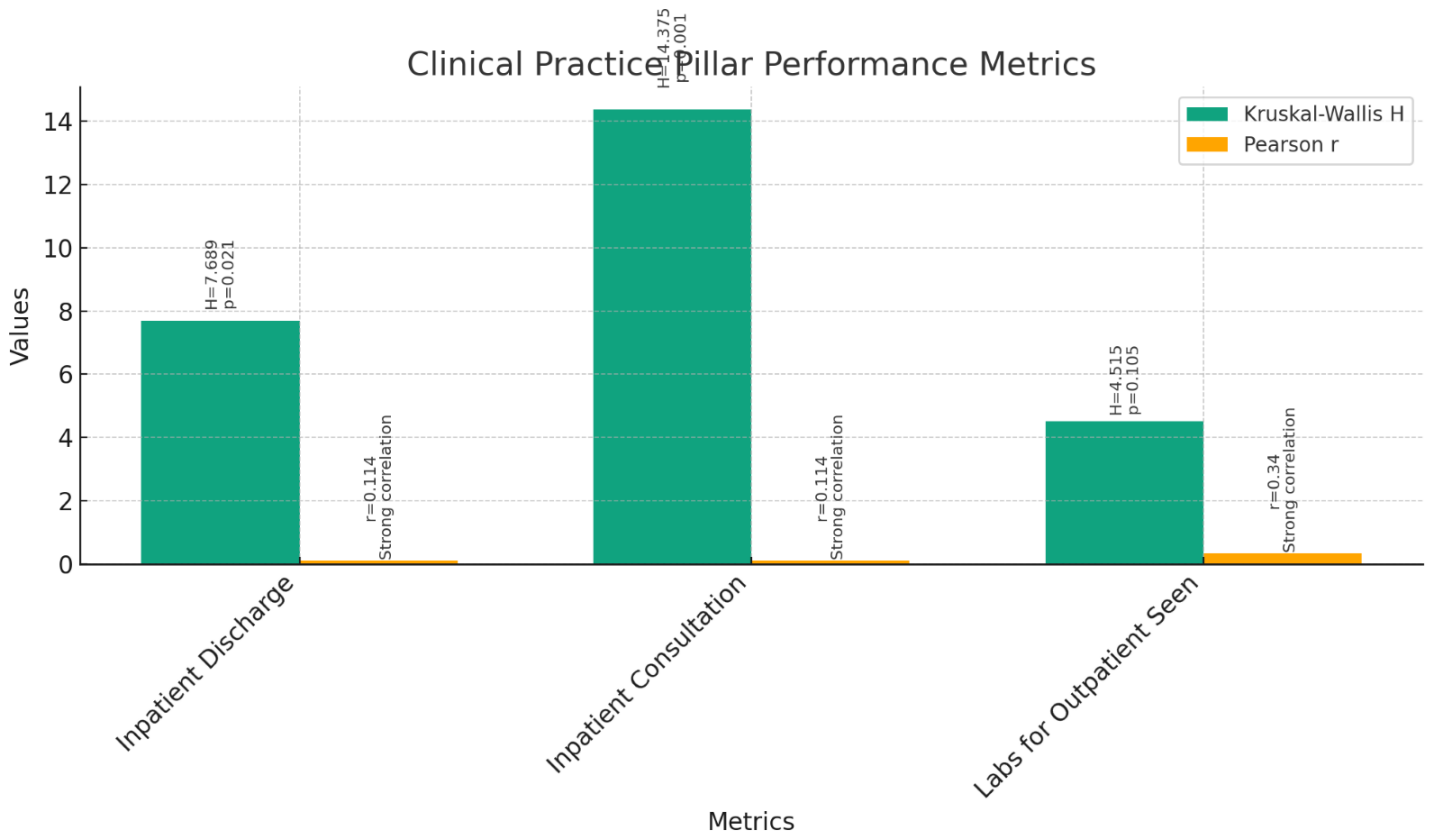
Clinical Practice Pillar Performance Metrics.

**Figure 7.**
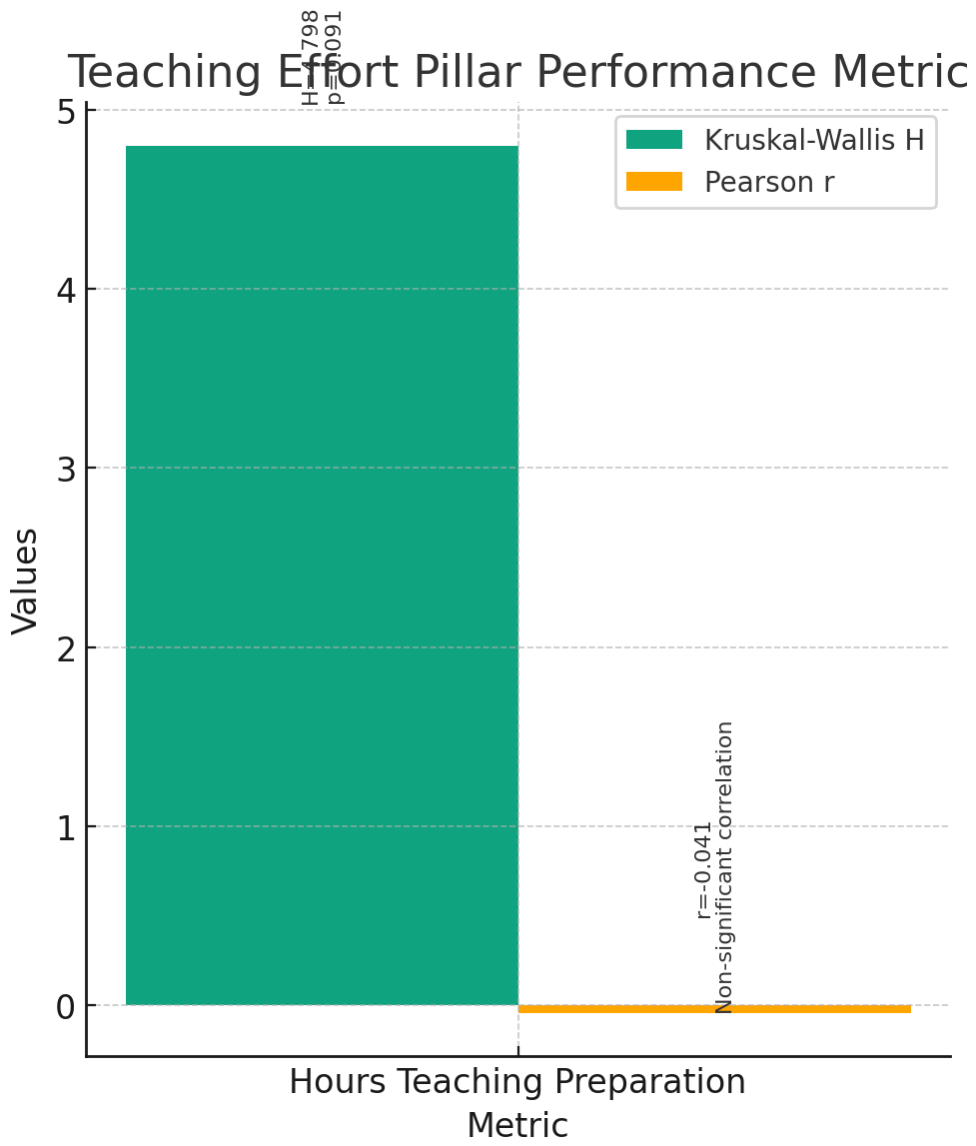
Teaching Effort Pillar Performance Metric.

**Figure 8.**
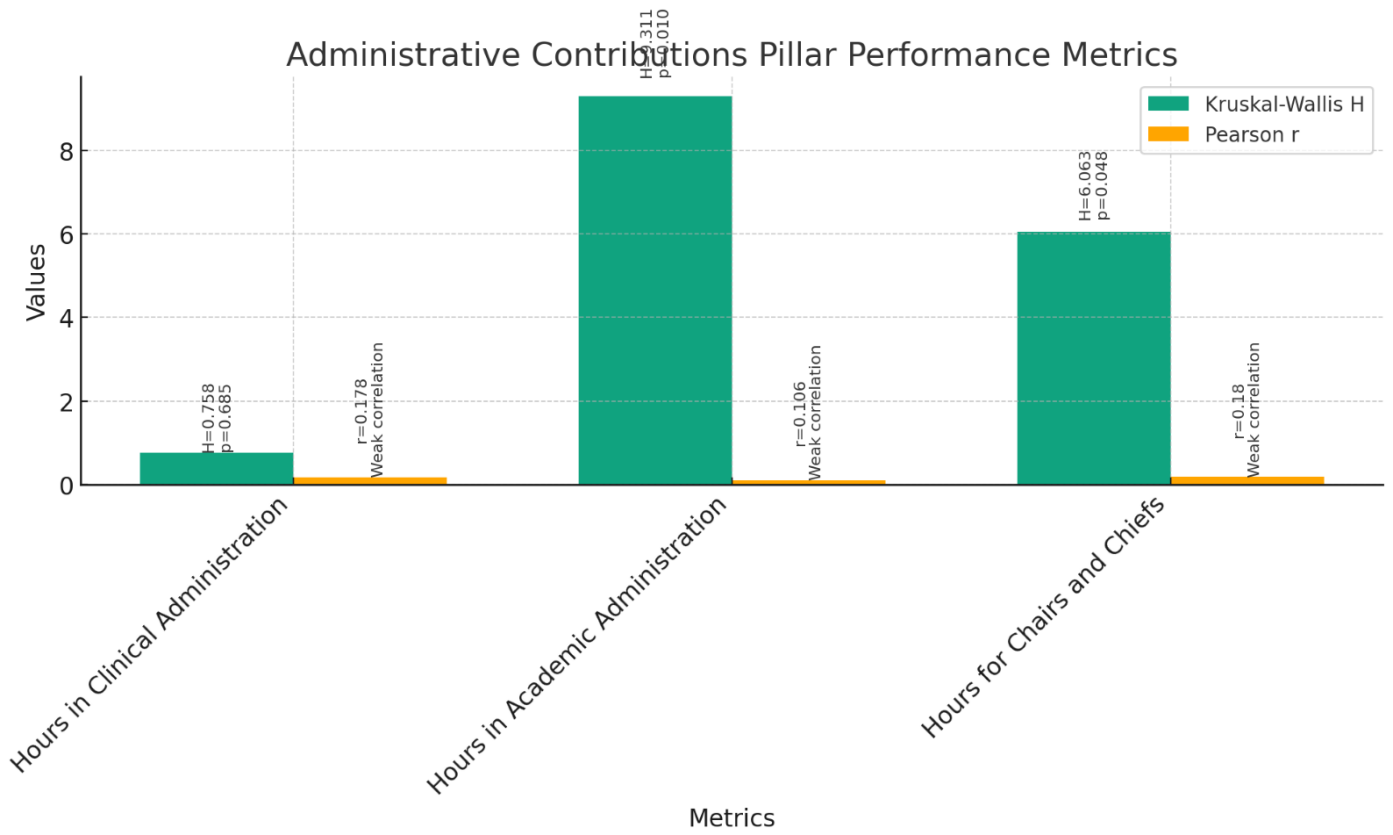
Administrative Contributions Pillar Performance Metrics.

**Figure 9.**
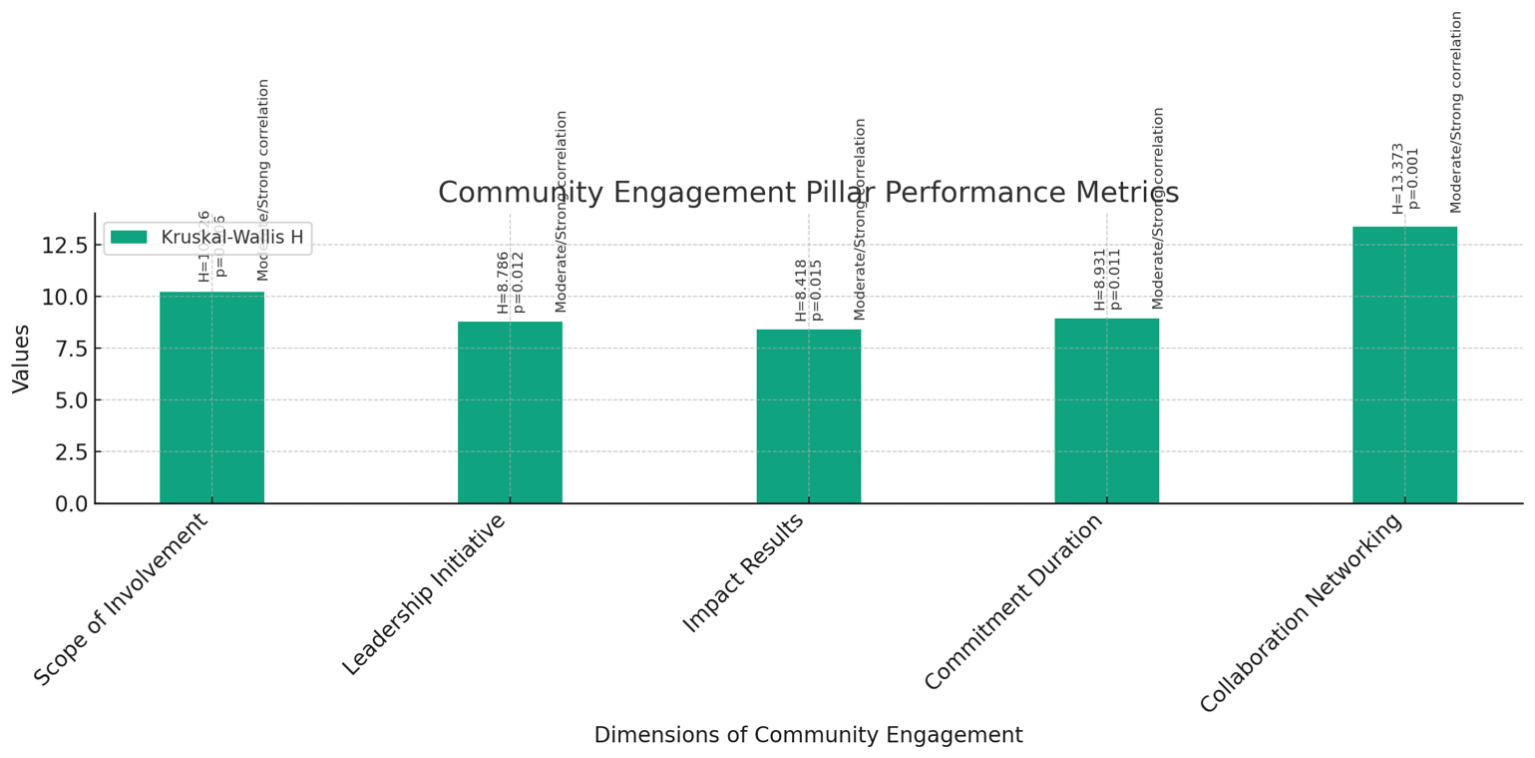
Community Engagement Pillar Performance Metrics.

**Figure 10.**
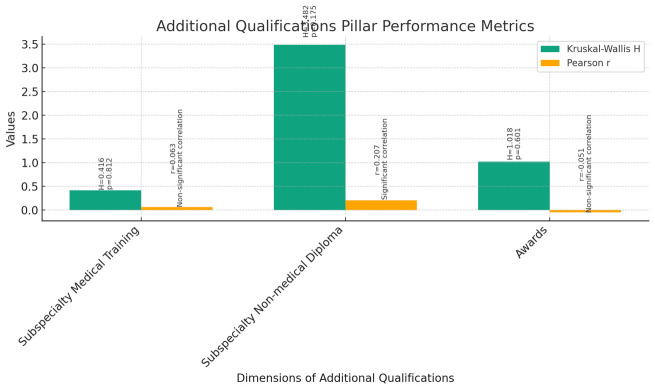
Additional Qualifications Pillar Performance Metrics.

- **Research Pillar ( Figure 5):** The Kruskal-Wallis test was conducted to compare the performance of instructors, assistant professors, and associate professors across the various metrics of the research pillar. The results indicated significant differences among the three groups for all metrics: Publications Total (H = 48.140, p < 0.001), Publications in the Last 7 Years (H = 11.810, p = 0.003), Adjusted Impact Factor per Author (H = 10.420, p = 0.005), Adjusted Impact Factor per Article (H = 6.588, p = 0.037), and H-Index (H = 37.548, p < 0.001).
  Further analysis through pairwise comparisons revealed that associate professors had significantly higher ranks in all metrics compared to instructors and assistant professors. Assistant professors also demonstrated higher ranks than instructors across all metrics, although the differences were less pronounced.
  Correlation analyses were conducted to explore the relationship between current rank and research productivity metrics. Pearson correlations showed significant positive correlations between current rank and Publications Total (r = 0.357, p < 0.01), and H-Index (r = 0.376, p < 0.01). Spearman's rho correlations confirmed these findings, indicating strong positive correlations between current rank and research productivity metrics (p < 0.01).
  These findings suggest that as faculty members progress in rank, their research productivity tends to increase. Associate professors, in particular, demonstrate higher levels of research output compared to instructors and assistant professors.
- **Clinical Practice Pillar ( Figure 6):** The Kruskal-Wallis test was conducted to compare the performance of instructors, assistant professors, and associate professors across various metrics within the Clinical Practice pillar. The results indicated significant differences among the three groups for Inpatient Discharge (H = 7.689, p = 0.021) and Inpatient Consultation (H = 14.375, p = 0.001), but no significance for Labs for Outpatient Seen (H = 4.515, p = 0.105).
  Further analysis through pairwise comparisons revealed that associate professors had significantly higher ranks in Inpatient Discharge and Inpatient Consultation compared to instructors and assistant professors. However, there were no significant differences between the groups for Labs for Outpatient Seen.
  Correlation analyses were conducted to explore the relationship between current rank and clinical practice metrics. Pearson correlations showed significant positive correlations between current rank and Inpatient Discharge (r = 0.231, p < 0.03) and Inpatient Consultation (r = 0.294, p < 0.01). However, no significant correlation was found between current rank and Labs for Outpatient Seen (r = 0.154, p > 0.1). Spearman's rho correlations confirmed these findings, indicating strong positive correlations between current rank and clinical practice metrics (p < 0.01).
  These findings suggest that as faculty members progress in rank, their involvement in clinical practice tends to increase, particularly in terms of inpatient activities. However, there is no significant difference in outpatient activities between different ranks.
- **Teaching Effort ( Figure 7):** The Kruskal-Wallis test was conducted to compare the performance of instructors, assistant professors, and associate professors across the metric of Hours Teaching Preparation at SGUB. The results indicated no significant differences among the three groups (H = 4.798, p = 0.091).
  Correlation analyses were conducted to explore the relationship between current rank and the metric of Hours Teaching Preparation at SGUB. Pearson correlation showed a non-significant negative correlation between current rank and Hours Teaching Preparation (r = -0.041, p = 0.666). Spearman's rho correlation also indicated a non-significant correlation (r = 0.064, p = 0.506).
  These findings suggest that there is no significant difference in the amount of time spent on teaching preparation among instructors, assistant professors, and associate professors. Additionally, there is no significant correlation between the current rank and Hours Teaching Preparation at SGUB.
- **Administrative Contributions ( Figure 8):** The Kruskal-Wallis test was conducted to compare the performance of instructors, assistant professors, and associate professors across various teaching administrative activities, including Hours in Clinical Administration, Hours in Academic Administration, and Hours for Chairs and Chiefs. The results indicated no significant differences among the three groups for Hours in Clinical Administration (H = 0.758, p = 0.685). However, significant differences were found for Hours in Academic Administration (H = 9.311, p = 0.010) and Hours for Chairs and Chiefs (H = 6.063, p = 0.048).
  Further examination through pairwise comparisons revealed no significant differences between instructors, assistant professors, and associate professors for Hours in Clinical Administration. However, significant differences were observed for Hours in Academic Administration and Hours for Chairs and Chiefs.
  Correlation analyses were conducted to explore the relationship between current rank and teaching administrative activities. Pearson correlations showed weak positive correlations between current rank and Hours in Clinical Administration (r = 0.178, p = 0.060), Hours in Academic Administration (r = 0.106, p = 0.267), and Hours for Chairs and Chiefs (r = 0.180, p = 0.058). Spearman's rho correlations confirmed these findings, indicating weak positive correlations between current rank and teaching administrative activities.
  These findings suggest that while there are no significant differences in Hours in Clinical Administration among instructors, assistant professors, and associate professors, there are significant differences in Hours in Academic Administration and Hours for Chairs and Chiefs. Additionally, current rank shows weak positive correlations with these teaching administrative activities, implying a slight increase in involvement as faculty members progress in rank.
- 5. **Community Engagement ( Figure 9):** The Kruskal-Wallis test was performed to assess the differences in community engagement activities among instructors, assistant professors, and associate professors across various dimensions, including Scope of Involvement, Leadership Initiative, Impact Results, Commitment Duration, and Collaboration Networking.
  The results revealed statistically significant differences among the three groups for all dimensions: Scope of Involvement (H = 10.226, p = 0.006), Leadership Initiative (H = 8.786, p = 0.012), Impact Results (H = 8.418, p = 0.015), Commitment Duration (H = 8.931, p = 0.011), and Collaboration Networking (H = 13.373, p = 0.001).
  Correlation analyses were conducted to explore the relationship between current rank and community engagement activities. Pearson correlations showed moderate to strong positive correlations between current rank and all dimensions of community engagement, including Scope of Involvement, Leadership Initiative, Impact Results, Commitment Duration, and Collaboration Networking. Similarly, Spearman's rho correlations confirmed these findings, indicating moderate to strong positive correlations between current rank and community engagement activities.
  These findings suggest that as faculty members progress in rank from instructors to associate professors, their involvement, leadership, impact, commitment, and collaboration in community engagement activities tend to increase significantly.
- **Additional Qualifications ( Figure 10):** The Kruskal-Wallis test was conducted to assess the differences in additional qualifications among instructors, assistant professors, and associate professors across various dimensions, including Subspecialty Medical Training, Subspecialty Non-medical Diploma, and Awards.
  The results indicated no statistically significant differences among the three groups for Subspecialty Medical Training (H = 0.416, p = 0.812), Subspecialty Non-medical Diploma (H = 3.482, p = 0.175), and Awards (H = 1.018, p = 0.601).
  Correlation analyses were performed to examine the relationship between current rank and additional qualifications. Spearman’s correlations revealed a significant positive correlation between current rank and Subspecialty Non-medical Diploma (r = 0.207, p = 0.028), indicating that as faculty members progress in rank, they tend to obtain more non-medical diplomas. However, there were no significant correlations found between current rank and Subspecialty Medical Training (r = 0.063, p = 0.507) or Awards (r = -0.051, p = 0.594).
  Spearman's rho correlations confirmed these findings, showing a significant positive correlation between current rank and Subspecialty Non-medical Diploma (rho = 0.196, p = 0.039), but no significant correlations were observed for Subspecialty Medical Training (rho = 0.055, p = 0.566) or Awards (rho = -0.095, p = 0.318).
  These results suggest that while there may be some association between current rank and the acquisition of non-medical diplomas, there are no significant differences in Subspecialty Medical Training or Awards among instructors, assistant professors, and associate professors.

To add robustness and validity to the cutoff scores used for academic promotions, it was essential to investigate the statistical differences within the same academic ranks between those who were promoted and those who were not. This approach aimed to identify and quantify the factors that distinctly contribute to successful promotions, thereby providing a more objective and transparent basis for setting these crucial benchmarks. Hence, the Kruskal-Wallis tests were employed across various academic ranks (instructors, assistant professors, and associate professors) to explore significant differences in a comprehensive set of metrics between promoted and non-promoted individuals.

The Kruskal-Wallis tests conducted across various academic ranks (instructors, assistant professors, and associate professors) revealed significant differences between promoted and non-promoted individuals within the same rank across a range of metrics. Instructors who received promotions demonstrated significant disparities in total publications (H = 13.008, p < 0.001), publications within the past seven years (H = 11.001, p = 0.001), adjusted impact factors per author (H = 8.040, p = 0.005), and per article (H = 6.340, p = 0.012). Further differences were noted in the H-Index (H = 3.669, p = 0.055), clinical practice (H = 5.504, p = 0.019), and hours dedicated to teaching preparation (H = 7.954, p = 0.005) and clinical administration (H = 4.982, p = 0.026). Leadership involvement (H = 9.873, p = 0.002), commitment duration (H = 8.710, p = 0.003), and collaboration and networking (H = 4.372, p = 0.037) also varied significantly among promoted instructors.

Similarly, assistant professors who received promotions exhibited significant disparities in total publications (H = 8.212, p = 0.004), adjusted impact factors per author (H = 4.871, p = 0.027), per article (H = 8.381, p = 0.004), and mean Z-scores for research (H = 8.735, p = 0.003). Variations were also observed in hours dedicated to teaching preparation (H = 11.832, p = 0.001), clinical administration (H = 7.108, p = 0.008), and academic administration (H = 9.313, p = 0.002). Moreover, differences in involvement in leadership initiatives (H = 13.310, p < 0.001), commitment duration (H = 6.071, p = 0.014), and collaboration and networking (H = 8.041, p = 0.005) were significant among promoted assistant professors.

For associate professors, significant differences were observed between those who were promoted and those who were not in total publications (H = 6.686, p = 0.010), publications within the past seven years (H = 9.075, p = 0.003), and mean Z-scores for research (H = 9.460, p = 0.002). Significant disparities also emerged in academic administration hours (H = 10.788, p = 0.001) and involvement in leadership initiatives (H = 12.349, p < 0.001), alongside commitment duration (H = 7.730, p = 0.005) and collaboration and networking (H = 3.031, p = 0.051).

In conclusion, our comprehensive analysis of the SGUB FM promotion matrix through the Kruskal-Wallis test and correlation techniques has revealed that promotions within our academic institution are influenced by a diverse set of factors, encompassing research output, impact factors, professional engagement in teaching, clinical practice, administration, and leadership roles. The complexity and breadth of these factors highlight the multifaceted nature of academic advancement, emphasizing that successful promotion relies not only on scholarly contributions but also on effective involvement in broader institutional responsibilities. Specifically, while associate professors often exhibit superior performance in research and clinical responsibilities, no substantial differences in teaching preparation time were found across ranks. Distinct variations in administrative contributions, particularly in academic administration and leadership roles, suggest a modest yet progressive increase in administrative engagement with advancing rank. Additionally, a noteworthy finding showed that community engagement intensifies with faculty progression, underlining the increasing involvement in broader academic and social initiatives. Conversely, additional qualifications such as non-medical diplomas displayed some rank-based associations, but medical training and awards did not demonstrate significant variability among ranks. This section underscores that as faculty members ascend the academic ladder at SGUB FM, their contributions diversify and intensify, reflective of the institution's commitment to fostering professional growth and recognizing multifaceted excellence.

## Discussion

### Validation of the promotion matrix and gender equity

The SGUB FM promotion matrix provides an objective and structured evaluation framework, ensuring fair and data-driven faculty assessments. Gender analysis confirmed that the promotion process is statistically representative of the faculty population, with no significant disparities in key performance metrics. While minor trends in clinical and administrative scores suggest historical role distributions, they do not indicate systemic bias. To further enhance equity, institutional efforts should focus on expanding leadership opportunities and fostering inclusive faculty development initiatives.

### Rank-based differences in faculty performance

The structured evaluation of faculty performance demonstrated that higher academic ranks align with increased research productivity, clinical engagement, and administrative contributions. These findings affirm that the promotion process at SGUB FM is aligned with expected faculty career progression patterns, ensuring merit-based advancements that reflect individual contributions to research, teaching, clinical service, and institutional leadership. Notably, teaching preparation time remained stable across ranks, indicating that instructional efforts are consistently valued regardless of seniority. Moving forward, targeted strategies should support faculty research and leadership development to sustain academic excellence.

### Differences within the same rank

Further validation of the promotion matrix revealed distinct performance patterns within faculty ranks. Promoted faculty consistently outperformed non-promoted peers in research output, clinical engagement, and leadership responsibilities. These differences reinforce the merit-based nature of the promotion system, ensuring that faculty members who demonstrate sustained excellence in multiple domains advance accordingly.

The findings also highlight the importance of a well-rounded faculty profile. While research productivity remains a critical factor, contributions in clinical practice, administrative roles, and community engagement play a significant role in distinguishing faculty members within the same rank. This underscores the necessity of a holistic evaluation framework that recognizes the multifaceted nature of academic contributions.

Moreover, these distinctions suggest that targeted support mechanisms, such as structured mentorship programs and faculty development workshops, can further enable professional growth. By providing early-career faculty with resources to strengthen their research capabilities, leadership skills, and institutional involvement, academic institutions can ensure a more equitable promotion process. Such initiatives not only facilitate career progression but also enhance overall faculty engagement and institutional success.

Further validation of the promotion matrix revealed distinct performance patterns within faculty ranks. Promoted faculty consistently outperformed non-promoted peers in research output, clinical engagement, and leadership responsibilities. This highlights the effectiveness of the matrix in distinguishing high-performing faculty and underscores the need for structured mentorship programs and research incentives to facilitate career advancement.

### Refined Weighting System for Promotion Matrix

A data-driven revision of the weighting system ensures that each evaluation pillar accurately reflects its contribution to faculty progression. Research remains the strongest predictor of promotion, while community work and extra degrees have been adjusted to reflect their empirical significance. Future iterations should incorporate qualitative assessments such as peer evaluations and interdisciplinary collaborations to create a more holistic framework.

### Limitations and future directions

While the findings support the validity of the SGUB FM promotion matrix, several limitations must be acknowledged. The study relies primarily on quantitative assessments, which, while ensuring objectivity, may not fully capture qualitative aspects such as mentorship, interdisciplinary collaboration, and leadership effectiveness ( Steinert *et al.*, 2012). Many promotion frameworks, including those recommended by the Association of American Medical Colleges ( AAMC, 2019), advocate for a more holistic evaluation, integrating qualitative peer evaluations, student feedback, and leadership impact, which are currently not included in this model.

Additionally, the weighting system, though refined using statistical analyses, applies fixed values that may not fully accommodate disciplinary variations across faculty members ( Xierali *et al.*, 2021). While the study effectively quantifies faculty performance, it does not account for specialty-based variations in clinical practice or the impact of non-traditional academic contributions such as educational innovation or public health initiatives. Future iterations of this model should consider flexible or discipline-specific weightings to improve adaptability.

Another key limitation is the cross-sectional nature of this study, which captures faculty performance at a single time point but does not track career progression, retention rates, or the long-term impact of promotion decisions. Longitudinal analyses could provide a stronger predictive framework for understanding how promotion criteria affect faculty trajectories ( Jacobs *et al.*, 2020). Future studies should incorporate long-term faculty tracking to evaluate the sustainability and effectiveness of promotion policies.

Furthermore, while gender equity was statistically supported, the study does not account for external factors influencing faculty productivity, such as institutional support structures or implicit biases in academic progression ( Carnes *et al.*, 2015). Research has shown that female faculty members may face greater service burdens, fewer mentorship opportunities, and disparities in research funding, all of which can influence long-term career advancement. To enhance the model’s applicability, future studies should explore intersectional analyses considering gender, discipline, and career stage.

Finally, the research is limited to a single institution, restricting its generalizability to other academic environments. Future studies should validate this framework in multi-institutional settings to determine whether similar patterns hold across diverse academic structures. Benchmarking against other promotion models from international medical schools (e.g., AAMC, AUB, NYU Grossman) will improve the model’s robustness and applicability beyond SGUB FM.

## Ethics and consent

This study was conducted with the written approval of the Institutional Review Board (IRB) at Saint George University of Beirut Faculty of Medicine (SGUB FM). The ethical approval was granted under the reference number IRB-REC/O/021-24/1124 issued on 19 June 2024.

### Participant consent

All participants in this study provided informed consent prior to their participation. The consent process was conducted as follows:

1. Type of Consent: Written consent was obtained from all participants.
2. Consent Process: Participants were provided with a detailed information sheet outlining the study's purpose, procedures, potential risks, and benefits. They were given the opportunity to ask questions and were assured of their right to withdraw from the study at any time without any consequences.

The ethical guidelines followed in this study adhered to the principles set forth in the Declaration of Helsinki and the regulations established by the SGUB FM IRB. The anonymity and confidentiality of the participants were maintained throughout the study, and all data were securely stored and accessed only by authorized personnel.

## Data Availability

Figshare: https://doi.org/10.6084/m9.figshare.25713831.v1 ( Jreij, 2024) This project contains the following underlying data: •   ARPP-SGUB Data are available under the terms of the Creative Commons Attribution 4.0 International license (CC-BY 4.0).
