# Supplementary material for: Implementing a multidimensional faculty promotion matrix at Saint George University of Beirut Faculty of Medicine
Source: MedEdPublish (2016). 2025 Apr 3;14:65. Originally published 2024 Aug 27. [Version 2] doi: 10.12688/mep.20416.2 (PMC12177446; doi:10.12688/mep.20416.2)
Supplement: Peer review history

# Reviewer report: Reviewer response for version 2

**DOI:** 10.21956/mep.22421.r41940

**Published:** 2025-06-19

**Authors:** Jamiu O Busari

**Affiliations:** 1 Maastricht University, Maastricht, The Netherlands; 2 Pediatrics, Horacio Oduber Hospital, Oranjestad, Aruba

The paper focuses on a critical aspect of career development and attempts to design a just system for faculty promotion. The authors begin by establishing a compelling and well-substantiated rationale for the need for a credible meritocratic system in academic medicine, and they propose a structured six-pillar matrix as the basis for their approach. 

 The conceptual framework, rationale, and the matrix design appear credible and promising. However, I have several concerns regarding the evaluation methodology and interpretation of findings. While the study relies primarily on quantitative metrics and psychometric analysis, it is important to consider whether the selected methods are best suited to capture the complexity of what is being assessed. In this case, the omission of qualitative assessments in the matrix may limit the robustness and validity of the tool.

 For example, within the clinical practice pillar, the use of indicators such as inpatient consultations, discharges, and number of outpatient laboratory tests may not adequately reflect the quality or complexity of clinical care. These metrics may disproportionately favor certain specialties or practice settings and overlook critical nuances such as case complexity, duration of care, and surgical versus non-surgical interventions. Concretely, how do the 3 metrics used adjust for the number, duration, and complexity of treatment encounters in different specialties? And also for Surgical versus nonsurgical encounters, for example? That in itself affects the validity of the matrix, whether we are truly measuring what we should to draw the conclusions being made.

 With regard to the gender equity analysis, the authors’ reliance on Kruskal-Wallis analysis to demonstrate fairness across gender groups is acknowledged. However, the conclusion that the matrix performs equitably may be somewhat premature and I would have expected a more cautious tone, bearing in mind that some gender related factors have indirect negative influences on the “measured” performance. A more nuanced interpretation is warranted, particularly given that systemic and often unmeasured factors—such as the impact of parental leave, childrearing responsibilities, and implicit bias within certain specialties—can influence performance metrics. For example, the impact of pregnancy and raising children has been found to have disproportionate impacts on the careers of women and the hidden biases and prejudices in certain specialties tend to hinder/delay their career advancement too. Were these considered or corrected for in the analysis? It would strengthen the paper if the authors acknowledged these potential limitations and considered whether any corrective adjustments were made.

 Additionally, the manuscript notes that SGUB Faculty of Medicine, being a newly established institution, opted not to incorporate tenure into its promotion cycle, aligning with its emphasis on capacity building. While this is a reasonable institutional stance, it would be helpful if the authors clarified the nature of the promotions under consideration and elaborated on how the matrix supports the institution’s goals for faculty development and progression in the absence of tenure.

 In conclusion, the authors are to be commended for their proactive and well-intentioned effort to build a fairer system of faculty advancement. The initiative is timely and aligns well with broader conversations about reforming academic evaluation. To further strengthen the manuscript, I would encourage the authors to adopt a more cautious tone in interpreting their findings and to expand the discussion and limitations sections to acknowledge some of the methodological and conceptual concerns raised above. With these refinements, the paper could make a valuable contribution to the literature on faculty promotion in academic medicine.

Is the case presented with sufficient detail to be useful for teaching or other practitioners?

Partly

Is the work clearly and accurately presented and does it cite the current literature?

Yes

If applicable, is the statistical analysis and its interpretation appropriate?

I cannot comment. A qualified statistician is required.

Is the case’s background and context in sufficient detail?

Yes

Are all the source data underlying the results available to ensure full reproducibility?

Yes

Are the conclusions drawn adequately supported by the results?

Partly

Reviewer Expertise:

Faculty development, Educational leadership, DEI

I confirm that I have read this submission and believe that I have an appropriate level of expertise to confirm that it is of an acceptable scientific standard, however I have significant reservations, as outlined above.

# Reviewer report: Reviewer response for version 2

**DOI:** 10.21956/mep.22421.r41943

**Published:** 2025-06-18

**Authors:** Rachmadya Nur Hidayah

**Affiliations:** 1 Department of Medical Education and Bioethics, Universitas Gadjah Mada, Yogyakarta, Special Region of Yogyakarta, Indonesia

The manuscript offers a promising topic by focusing on the faculty promotion as an emergent issue relevant to medical and health professions education in general.

 The authors should consider improving the background by providing a deeper contextualization. Specifically, elaborating on any pre-existing promotion frameworks under the University of Balamand before SGUB FM’s establishment would enhance the foundational narrative. More precisely articulating the specific limitations in faculty evaluation that the proposed matrix aims to address would provide a more explicit rationale for its development.

 The authors should also include how faculty promotion has been implemented/ reported as frameworks worldwide (e.g., citing the roles of medical teachers, academic promotion requirements, etc.) to give a more balanced view on the discourse. These improvements would strengthen the manuscript’s contextual framing and justification.

 The manuscript effectively outlines the development and structure of the faculty promotion matrix, including its reliance on six performance pillars and z-score calculations for evaluation. Although the authors proposed a well-structured matrix, the justification for the criteria can be written more clearly with stronger arguments. The focus of the matrix is predominantly on performance metrics rather than direct outcomes of promotion decisions, which undermines the claim of equitable promotion rates across ranks. More explanation should be given on how the consensus agreement is designed and conducted. 

 The lack of operational details limits the manuscript’s utility for teaching or application by other institutions. Although the development process is briefly described, the manuscript does not provide sufficient replication details. For example, insights into challenges faced during the matrix’s implementation and how faculty feedback informed the design would enhance its pedagogical and practical relevance. The context of faculty leadership (including leadership, role modelling, and mentorship in management) may enrich the framework’s utility. Again, referring to the broader literature of faculty promotion and development programs would benefit the discussion section. 

 Authors may consider adding several key references as listed in the citation. Leslie K 2014, Morrison L et. al 2014, Steinert Y et. al 2012, Tung J et. al 2021 (Ref 1,2,3 and 4)

 The alignment between the data, discussion, and conclusion should be more palpable. A more detailed explanation of how the data supports the findings could improve the manuscript’s transparency and facilitate replication or secondary analysis by researchers.

Is the case presented with sufficient detail to be useful for teaching or other practitioners?

Partly

Is the work clearly and accurately presented and does it cite the current literature?

Partly

If applicable, is the statistical analysis and its interpretation appropriate?

I cannot comment. A qualified statistician is required.

Is the case’s background and context in sufficient detail?

Partly

Are all the source data underlying the results available to ensure full reproducibility?

Partly

Are the conclusions drawn adequately supported by the results?

Partly

Reviewer Expertise:

Medical and health professions education, assessment, clinical simulation, quality assurance

I confirm that I have read this submission and believe that I have an appropriate level of expertise to confirm that it is of an acceptable scientific standard, however I have significant reservations, as outlined above.

## References

[1] : Faculty Development for Academic and Career Development.2014; 10.1007/978-94-007-7612-8_5 97-118 10.1007/978-94-007-7612-8_5

[2] : Impact of a formal mentoring program on academic promotion of Department of Medicine faculty: A comparative study. *Medical Teacher* .2014;36(7) : 10.3109/0142159X.2014.899683 608-614 10.3109/0142159X.2014.899683 24804918

[3] : Faculty development initiatives designed to promote leadership in medical education. A BEME systematic review: BEME Guide No. 19. *Medical Teacher* .2012;34(6) : 10.3109/0142159X.2012.680937 483-503 10.3109/0142159X.2012.680937 22578043

[4] : The impact of a faculty development program, the Leadership in Academic Medicine Program (LAMP), on self-efficacy, academic promotion and institutional retention. *BMC Medical Education* .2021;21(1) : 10.1186/s12909-021-02899-y 10.1186/s12909-021-02899-y 34474670 PMC8414755

# Reviewer report: Reviewer response for version 2

**DOI:** 10.21956/mep.22421.r41746

**Published:** 2025-06-05

**Authors:** Ardi Findyartini

**Affiliations:** 1 Universitas Indonesia, Depok, West Java, Indonesia

The paper titled 'Implementing a multidimensional faculty promotion matrix at Saint. George University of Beirut Faculty of Medicine' has been revised and further reviewed. The comprehensive approach in developing the multidimensional faculty promotion is excellent. While the overall paper really emphasize the essential pillars (research, clinical practice, teaching effort, administrative effort, and community work), I would recommend stronger emphasis and discussion on teaching effort pillar. 

 a. The introduction elaborated that the faculty promotion regarding teaching and education should be aligned with curriculum needs. This is particularly appropriate, yet it is not clear which context/continuum of curriculum being referred to. I would recommend more detailed arguments on this involving the context of undergraduate and postgraduate curricula where appropriate, according to the context of the current faculty of medicine. 

 b. I would recommend using and discussing the expanded definition of scholarship by Boyer and Glassick in the introduction and  to further inform the pillars and matrices.

 c. Teaching effort pillar consists of teaching hours and curriculum development contribution. How do these resources of data of faculty performance also include teaching/supervision in different contexts (in class, in large or small groups, in simulation setting, in bedside, in research etc), and involvement in conducting assessment and providing feedback etc. I also believe that contributions in curriculum development should also include scholarship of teaching and learning and contribution in different forms of faculty development programs in the institution.

Is the case presented with sufficient detail to be useful for teaching or other practitioners?

Yes

Is the work clearly and accurately presented and does it cite the current literature?

Partly

If applicable, is the statistical analysis and its interpretation appropriate?

I cannot comment. A qualified statistician is required.

Is the case’s background and context in sufficient detail?

Yes

Are all the source data underlying the results available to ensure full reproducibility?

Partly

Are the conclusions drawn adequately supported by the results?

Partly

Reviewer Expertise:

Faculty development, curriculum development, professionalism

I confirm that I have read this submission and believe that I have an appropriate level of expertise to confirm that it is of an acceptable scientific standard, however I have significant reservations, as outlined above.

# Reviewer report: Reviewer response for version 2

**DOI:** 10.21956/mep.22421.r41341

**Published:** 2025-04-18

**Authors:** Shivasakthy Manivasakan

**Affiliations:** 1 Sri Balaji Vidyapeeth, Puducherry, India

It's a well-conducted study with proper analysis of data. The manuscript is well written and elaborately cites relevant literature evidence. The outcome of the study on the development and implementation of a multidimensional faculty promotion matrix designed to provide a comprehensive and equitable evaluation of faculty across multiple dimensions will be helpful for future implementation in other institutions.

Is the case presented with sufficient detail to be useful for teaching or other practitioners?

Yes

Is the work clearly and accurately presented and does it cite the current literature?

Yes

If applicable, is the statistical analysis and its interpretation appropriate?

Yes

Is the case’s background and context in sufficient detail?

Yes

Are all the source data underlying the results available to ensure full reproducibility?

Yes

Are the conclusions drawn adequately supported by the results?

Yes

Reviewer Expertise:

Health Professions Education

I confirm that I have read this submission and believe that I have an appropriate level of expertise to confirm that it is of an acceptable scientific standard.

# Reviewer report: Reviewer response for version 1

**DOI:** 10.21956/mep.21848.r40304

**Published:** 2024-12-17

**Authors:** Xiang Zheng

**Affiliations:** 1 University of Wisconsin-Madison, Madison, USA

This article examines the practices of developing an indicator matrix for faculty promotion at the medical department of a Lebanese university. I appreciate the inclusion of diverse criteria such as teaching, clinical activities, administrative work, and community contributions, which are often undervalued in other countries and universities. This study has merit, but to make the findings more transferable and actionable to an international audience, the article needs to increase clarity and rigor on the measures, data sources, weighting rationale, and validation methods.

1.  In the introduction or literature review, the authors should investigate and benchmark their indicator matrix against promotion criteria used by universities worldwide. This step is necessary to identify best practices, gaps, and areas of innovation and to ensure significance and relevance to the global academic community. This would allow the authors to demonstrate how their matrix addresses existing limitations and provide unique implications.
2. The article assigns weights to indicators without a theoretical foundation or justification. For example, I don’t understand why prioritizing the H-index over impact factors or recent publications. The total publications, recent publications, and H-index should be highly correlated, but they are assigned with separate weights. Similarly, it strangely assigns lower weights to time spent in chief administrative roles at both workplaces compared to other single-workplace positions, and I also think they are correlated. The lack of scientific methods, such as the Delphi technique, undermines the credibility of the weighting process.
  The evaluation framework lacks specificity in several areas:
    - Teaching: The evaluation of teaching is always a challenge in higher education evaluation. However, no clear itemized criteria or methodology for assessing teaching quality are provided in this study, limiting its contribution to education evaluation.
    - Clinical and Administrative Work: The matrix only considers workload or investment (e.g., patient numbers, hours worked) but overlooks the resulting output, work feedback, and measurable achievements.
    - Community Contributions: The key areas for evaluation are not well-defined, and there is no explanation of how they were determined, selected, assessed, and quantified.
   - Including Extra Medical Degrees and Non-Medical Degrees as indicators seems misplaced and does not effectively reflect faculty output or achievement.
3. Many data sources used in the matrix are self-reported information and personal CVs, which are prone to self-selection bias and may compromise the reliability of the results. Existing studies have shown that some groups of people may tend to exaggerate their achievements in CVs.
4. The threshold score of 50 for promotion eligibility appears arbitrary and lacks empirical or theoretical justification, which is concerning given its significance for career advancement. The authors should test the results’ sensitivity to different thresholds.
5. The results part, which compares promotion rates and indicator performance across ranks, is disconnected from the original goal to establish that the matrix accurately reflects individual performance. This logically can’t validate the indicator matrix but only reflect some rank-wise differences. To strengthen the validation, the authors should benchmark their matrix against established evaluation frameworks, base it on robust theoretical frameworks, and use some data external to the framework to test correlations. For example, they could review peer evaluation comments to verify whether high-scoring faculty receive positive feedback.

Is the case presented with sufficient detail to be useful for teaching or other practitioners?

No

Is the work clearly and accurately presented and does it cite the current literature?

No

If applicable, is the statistical analysis and its interpretation appropriate?

No

Is the case’s background and context in sufficient detail?

No

Are all the source data underlying the results available to ensure full reproducibility?

No

Are the conclusions drawn adequately supported by the results?

No

Reviewer Expertise:

science of science, scientometrics, higher education evaluation

I confirm that I have read this submission and believe that I have an appropriate level of expertise to state that I do not consider it to be of an acceptable scientific standard, for reasons outlined above.

# Reviewer report: Reviewer response for version 1

**DOI:** 10.21956/mep.21848.r39191

**Published:** 2024-10-29

**Authors:** Laura Naumann

**Affiliations:** 1 Nevada State University, Henderson, USA

1. There are many fundamental flaws with this paper that prevent me from recommending this for indexing.  The first fundamental flaw is that this study purports to offer a new approach to evaluating Medical School Faculty in a fair and inclusive way. However, the authors’ Introduction is sparse and cites little previous work discussing current issues with what is valued for promotion and the disproportionate impact on women and racial minorities. 
2. Even if there weren’t a myriad of methodological and statistical problems (detailed further below), the authors conducted no statistical analyses showing how their proposed matrix eliminates gender or racial bias in promotion. For example, I believe they had hoped to make a case that their matrix is more inclusive because it credits faculty for their clinical work; however, they present no analyses illustrating how this approach would result in promotion for a predominately clinical faculty member. The authors briefly describe a categorization grouping – prefixes vs. suffixes – which seem related to research vs. clinical faculty, but there are no further statistical analyses using these categories. 
3. Authors did not discuss how they came to choose the various weights (%) that they did as part of them. Was this grounded in AAMC criteria or just their own thinking of what is valued at their institution?
4. It was not clear how Teaching Effort was calculated on page 5; later clarified as hours taught on page 11.  This should be explicated earlier
5. Authors do not provide enough detail to replicate calculation for Community Work.  How were things coded from faculty CVs and given numeric values?
6. Many problems with statistical analyses.  T-scores, not Z-scores, are normed to have a mean of 50 and a standard deviation of 10. Z-scores typically range from -3 to +3 and have a Mean of 0.  A person with a z-score of 0 would be “at the mean” or considered average. If you want to “norm” each of your criteria, you’d need to generate a table of means and standard deviations across your entire sample of 112 faculty.  (e.g., M and SD for total publications; h-index, etc).  You’d then compute z-scores for each individual in the sample on a given criteria.  Then, you could “weight” each individual’s z-score in that category and then create a sum total of all weighted z-scores to get their “promotable score”. 
7. Even if you did those things, I’d still have grave concern because of your sampling issues and likely introducing gender bias into your norms (e.g., male publication bias). Furthermore, subdividing your sample into those above 50 points as “promoted” vs. under 50 points as “not promoted” is additionally problematic. How do your account for all of the associate professors who were promoted but you’ve labeled as not promotable?  How does this alleviate inequity?
8. In terms of Section II analyses, you are trying to conduct a correlation using categorical ranks… why not dummy code your ranks or just conduct means comparisons?
9. Discussion does not address limitations.  Sample is a huge limitation; authors do not discuss issue of self-selection bias (e.g., people who agreed to participate may publish more and would inflate means).  Authors also do nothing to compare the sample they did collect to the total population of medical faculty at their institution.  Were the rates of rank and gender comparable to those in the total population? Their sample appears to be predominately male.
10. I wish I could be more supportive of this work. I think there is value in attempting to make objective the faculty review process; even quantifying certain elements to generate a total score could be valuable.  However, the authors have not provided enough detail on how they came to their weighting decisions (presumably with an eye toward equity) nor have they demonstrated how their matrix would still result in promotion for clinically oriented faculty.

Is the case presented with sufficient detail to be useful for teaching or other practitioners?

No

Is the work clearly and accurately presented and does it cite the current literature?

Partly

If applicable, is the statistical analysis and its interpretation appropriate?

No

Is the case’s background and context in sufficient detail?

No

Are all the source data underlying the results available to ensure full reproducibility?

Yes

Are the conclusions drawn adequately supported by the results?

No

Reviewer Expertise:

quantitative research; psychology; workload equity

I confirm that I have read this submission and believe that I have an appropriate level of expertise to state that I do not consider it to be of an acceptable scientific standard, for reasons outlined above.
